# Multi-view clustering for multi-omics data using unified embedding

**DOI:** 10.1038/s41598-020-70229-1

**Published:** 2020-08-12

**Authors:** Sayantan Mitra, Sriparna Saha, Mohammed Hasanuzzaman

**Affiliations:** 1 Department of Computer Science, Indian Institute of Technology Patna, Bihta, Bihar 801103 India; 2 ADAPT Centre, Cork Institute of Technology, Cork, Ireland

**Keywords:** Computational biology and bioinformatics, Machine learning

## Abstract

In real world applications, data sets are often comprised of multiple views, which provide consensus and complementary information to each other. Embedding learning is an effective strategy for nearest neighbour search and dimensionality reduction in large data sets. This paper attempts to learn a unified probability distribution of the points across different views and generates a unified embedding in a low-dimensional space to optimally preserve neighbourhood identity. Probability distributions generated for each point for each view are combined by conflation method to create a single unified distribution. The goal is to approximate this unified distribution as much as possible when a similar operation is performed on the embedded space. As a cost function, the sum of Kullback-Leibler divergence over the samples is used, which leads to a simple gradient adjusting the position of the samples in the embedded space. The proposed methodology can generate embedding from both complete and incomplete multi-view data sets. Finally, a multi-objective clustering technique (*AMOSA*) is applied to group the samples in the embedded space. The proposed methodology, Multi-view Neighbourhood Embedding (*MvNE*), shows an improvement of approximately 2−3% over state-of-the-art models when evaluated on 10 omics data sets.

## Introduction

Modern data sets are usually comprised of multiple distinct feature representations, often referred to as multi-view data, providing consistent and complementary information^1^. For example, in the case of multilingual data, each language represents a separate view; in a biomedical data repository, a clinical sample record^2^ may include patient information, gene expression intensity and clinical traits etc. By exploiting the characteristics of different views, multi-view learning can obtain better performance over single view learning^1^. Multi-view clustering provides a natural way of generating clusters from multi-view data and has attracted considerable attention.

Multi-view learning has started with Canonical correlation analysis (CCA)^3^ and a series of works on co-training methods^4–7^. Co-training maximizes the mutual agreement amongst different views in a semi-supervised setting. The reasons of its success have been investigated by^8^ and^9^. According to the mechanisms and principles, multiview clustering methods can be broadly divided into four typical classes; (i) *subspace-based: * these models learn a unified feature representation from all the views.^10–16^; (ii) *late fusion based: * model under this category combines the clustering results from multiple views to obtain the final clustering^16–18^; (iii) *co-training based: * methods under this category treats multi-view data by using co-training strategy; (iv) *spectral based: * under this category, methods learn an optimal similarity matrix to capture the structure of the clusters, which serves as an affinity matrix for spectral clustering^19–21^.

Amongst these wide variety of multi-view clustering methods, subspace ones perform better and are widely studied. They attempt to exploit the subspace by adopting different techniques, including canonical correlation analysis (CCA)^22–25^, aims at finding linear projections of different views with maximal mutual correlation, structured sparsity^26^, Gaussian process^27,28^, kernel embedding^29^ and non-negative matrix factorization (NMF)^30,31^. These embedding techniques learn the latent feature set either by using co-variance or affinity matrix, or, by directly projecting or factorizing the feature sets to desired latent space, ignoring the data similarity ranking. Cao et al. proposed Diversity-induced Multi-view Subspace Clustering (DiMSC)^32^ that exploits the complementary information from different views. For enforcing diversity the algorithm uses Hilbert-Schmidt Independence Criterion (HSIC). Xie et al. proposed^33^, a tensor-Singular Value Decomposition (t-SVD) based multiview subscpace clustering. It uses tensor multi-rank to capture the complementary information between the views and solve the multi-view clustering problem as an optimization problem. Zhang et al.^34^ proposed two variations of Latent Multi-View Subspace Clustering (LMSC) algorithm, linear LMSC (lLMSC) and generalized LMSC (gLMSC). lLMSC uses a linear correlation between each view and the latent representation, and gLMSC uses neural networks to obtain the generalized relationships between the views.

Clustering has many important applications in the field of biology and medicine^35^. The rapid development of high throughput technology makes available a large number of different omics data to study the biological problems^36^. It is possible to collect multiple omics data for the same person. Through examining the different omics data, it is possible to distinguish reliably between different categories of cancer^37^. Integration of multiple omics data can better understand the underlying molecular mechanism in complex biological processes, and therefore offers more sophisticated ways to address biological or medical issues^38,39^. Thus, compared to single data types, multi-omics methods achieve better performance. So far, a lot of approaches for data integration have been suggested in the literature. These data integration methods mainly depend on two strategies: (i) space projection method^40^, and (ii) metric (similarity measures) fusion technique^41^.

Nevertheless, these methods follow very different approaches to obtain the patterns of samples or genes from multiple data domains. Earlier methods have utilized high correlated samples in the datasets to identify the multi-dimensional genomic modules^42–44^. However, these “co-modules” can only detect the sample structures across data types and may lead to biased clustering^45^. Shen et al. proposed iCluster which obtains the cluster-structures from multi-omics data by using a joint latent variable template. Mo et al. developed iClusterPlus^46^, the iCluster extension, which uses linear regression models to learn different properties of omics data. However, the major drawback of this method is that it holds some strong assumptions which may fail to capture meaningful biological information. SNF (similarity network fusion)^41^ can resolve such problems as an almost assumption-free and rapid approach and uses local structure preservation method to modify sample similarity networks for each type of data. But, SNF can only characterize pair-wise similarity (e.g., Euclidean distance) in the samples, and is sensitive to local data noises or outliers. Further, pair-wise similarity can’t capture the true underlying structure in different subspaces, leading to inaccurate clustering. Nguyen et al. proposed PINS^47^, to identify clusters that are stable in response to repeated perturbation of the data. It integrates clusters by examining their connectivity matrices for the different omics data. Mitra et al.^48^ proposed an ensemble-based multi-objective multi-view algorithm for classifying patient data. This method is computationally very expensive. One drawback common to all these algorithms is that they treat all omics equally, which may not be biologically appropriate. As a result, the clusters discovered are often poorly associated with patient outcomes. Thus, there is a scarcity of more effective integration approach.

**Figure 1:**
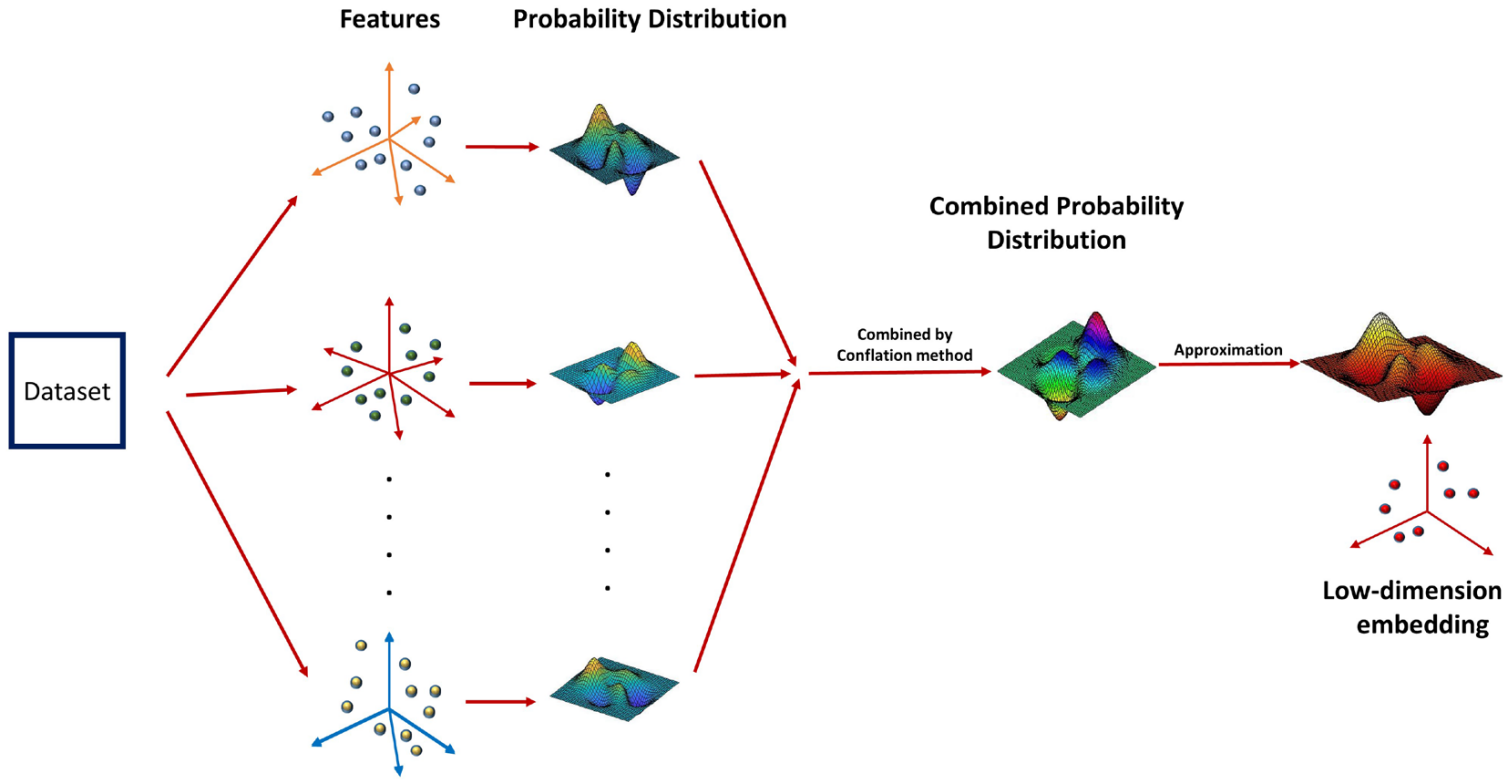
Different views of the data sets are combined in the probabilistic space by conflation method. The low-dimensional embedding is generated by approximating the combined probability distribution in the lower-dimensional space.

For patient stratification, multiple omics data can unfold more precise structure in the samples, that are not possible to disclose using single omic data. Combined information from multiple omics improves the performance of the clustering algorithm. Some of the advantages of using multi-omics clustering are as follows: (i) it reduces the effect of noise in the data, (ii) each omic can reveal structures that are not present in other omics, (iii) different omics can unfold different cellular aspects.

Motivated by the above requirements, in this paper, we have proposed a probabilistic approach to map the high dimensional multi-omics data to a low dimensional unified embedding preserving the neighbourhood identity across the views. It is meaningful to obtain an integrated heterogeneous feature set in a probabilistic model because different properties of the data, like, variance and mean, can be combined effectively in a probability space. Under each view in the higher dimensional space, a Gaussian is centered on every sample, and the densities under this Gaussian are used to generate a probability distribution over all the potential neighbours of the sample. The different probability distributions of each sample across different views are combined by conflation^49^. The aim is to approximate this unified distribution as much as possible when a similar operation is carried out in the embedded domain. Intuitively, a probabilistic embedding framework is a more conscientious approach because it circumvents the problems of different representations and incomparable scales. Further, we have applied multi-objective clustering to cluster the obtained embedded data sets. The main advantage of this technique is that it is capable of extracting different shaped clusters present in a data set. The general overview of the proposed methodology is shown in Fig. 1.

The proposed model *MvNE* (Multi-view Neighbourhood Embedding) is evaluated on 10 cancer data sets and results are compared with state-of-the-art methods.

Some of the benefits of the proposed methodology are as follows:

1. MvNE combines the views in the probability space. Combination of the views in the probability space preserves various statistical properties of the individual views.
2. Conflations of normal distributions coincide with the classical weighted least squares method, hence yielding best linear unbiased and maximum likelihood estimators. The use of this method provides a weighted combination of several views which is an important criterion for view combination. Hence, it reduces the overhead of finding optimal weights for each view.
3. The proposed methodology is extended to handle the datasets having incomplete views.
4. To the best of our knowledge, the current work is the first attempt in combining multiple omics data in the probability space in biomedical domain.
5. To the best of our knowledge, conflation method for combining multiple views was never used in the literature.

## Methods

Under this section, we have described the process of generating unified embedding to handle the multi-view data sets.

### Problem statement

Given a data set $\mathbf {X = \{x_1,x_2,\dots , x_n\}},$ with *n* samples, we use ${\mathbf {X}}^v=\{x^{v}_{1},x^{v}_{2},\dots ,x^{v}_{n}\}\in {\mathbb {R}}^{d_v \times n}(v=1,2,\dots , m)$, to represent the $v^{th}$ view of the data set with $d_v$ feature dimensions. The task is to obtain an embedding in the lower dimension, ${\mathbf {Y}}= \{\mathbf {y}_\mathbf {1},\mathbf {y}_\mathbf {2},\dots , \mathbf {y}_\mathbf {n}\}\in {\mathbb {R}}^{d_{emb} \times n}$, by unifying all *m* number of views, and categorizing it into *C* classes. Here, ${\mathbf {Y}}$ is optimized so that the sum of the Kullback-Leibler divergence between the two distributions (computed from higher dimension, ${\mathbf {X}}$, and lower dimension, ${\mathbf {Y}}$) is minimized.

### Conflation of probability

The conflation is defined as the distribution determined by the normalized product of the probability density or probability mass functions^49^. It can be easily calculated and also minimizes the maximum loss in Shannon information in combining several independent distributions into a single distribution. The conflation of normal distributions produces the classical weighted mean squares and the maximum likelihood estimators for normally-distributed unbiased samples^49^. The traditional methods of combining probability distributions, viz., averaging the probabilities and averaging the data, are as follows:

**Figure 2:**
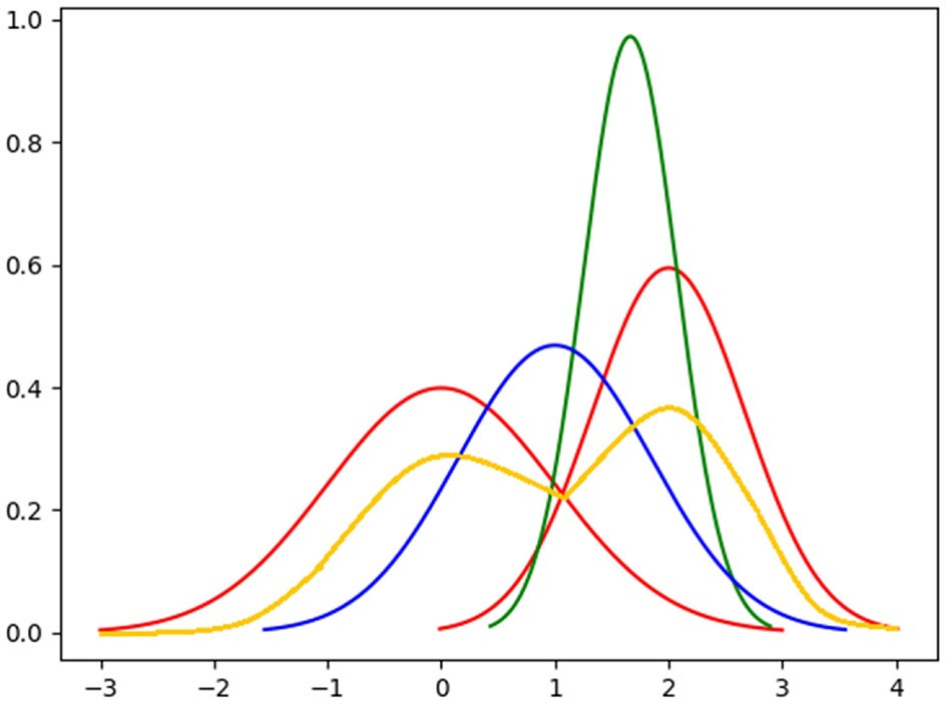
Example of conflation technique. The red curves are the two independent distributions, yellow curve is the probability distribution obtained by averaging the probabilities, blue curve is the probability distribution obtained by averaging the data and green curve denotes the distribution obtained by conflation technique.

#### Averaging the probabilities

One of the common methods of combining the probabilities is by averaging them over every set of values, $P(X) = (P_1(X)+P_2(X))/2$. This method has a significant disadvantage. Firstly, the mean of the combined distribution is always exactly the average of the means, independent of the relative accuracy or variance of each. It is unreasonable to weight the two distributions equally. Secondly, it generates a multi-modal distribution, whereas the desired output distribution should be in the form as that of the input data—normal, or at least unimodal.

#### Averaging the data

Another common method that does preserve the normality is to average the data. Here *P* is calculated on $(X_1 + X_2)/2$. Again, the main disadvantage of this method is that the obtained distribution has the mean exactly the average of the means of the input distributions, irrespective of the relative accuracies or variances of the two inputs (shown in Fig. 2). The variance of *P* is never larger than the maximum variance of $P_1$ and $P_2$.

The conflation of probabilities (denoted by symbol “&”) is a method for consolidating uniformly weighted data.

If $P_1$ and $P_2$ have probability mass functions of $f_1$ and $f_2$, respectively, then conflation is denoted as follows:

1 $$ \begin{aligned}{} \& (P_1,P_2) = \frac{f_1(x)f_2(x)}{\sum _y f_1(y)f_2(y)} \end{aligned}$$

In Fig. 2, we have shown the comparison between conflation, averaging the probabilities and averaging the data methods. Initially, it may seem counter-intuitive that conflation of the two distributions produces a much narrower curve. However, if the two measurements obtained from different sources are assumed equally valid, then the overlap region between the two distributions contains the real value with relatively high probability.

### Generation of initial unified data set by combining all views

Initially, we generate a unified data set, $X \in {\mathbb {R}}^{d \times n}$ by concatenating the views, $X^v \in {\mathbb {R}}^{d_v \times n}$, such that $d = \sum _vd_v$. For the points not appearing in all the views, we have replaced the missing features with zeros.

After obtaining *X*, we have used a stacked autoencoder (SAE) to obtain an unified representation of the data set, $Y_{init} \in {\mathbb {R}}^{d_{emb} \times n}$. Here, $d_{emb}$ represents the feature dimension in the embedded domain. Recent research has shown that SAE consistently produces well separated and semantically meaningful representation on real-world data^50^.

**Figure 3:**
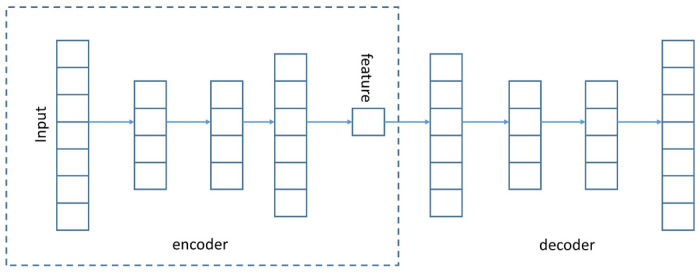
Network structure of the stacked autoencoder. Output of the “feature” layer is the $\mathbf {Y_{init}}.$

The SAE comprises of an autoencoder part and a decoder part. Suppose, we are having a three layer architecture: an input layer, three hidden layers and an output layer. In Fig. 3, the “feature” layer is the bottleneck i.e., the output of this layer is the required embedding. In the input layer, we provide the original sample vector as input. For example, we provide a vector of size 200 as input and want the embedding to be of size 30. Then the input and output layers will have size 200 and the bottleneck layer will have size, 30. The input vector is first squeezed to the size of 30 and then we reconstruct the 200 sized vector from this size of 30. The reconstructed vector, i.e., the value of the output layer should be similar to the input vector. For this, we have used mean squared error between the input and output vectors as the loss function. Once the network is trained, the decoder part is discarded and only the encoder part is used to generate the embedding. The details of the network is explained below.

Each layer of SAE is a denoising autoencoder, trained to reconstruct the output of the previous layer after random corruption^50^.

The denoising autoencoder is defined as follows:

2 $$\begin{aligned}&{\hat{x}} \sim Dropout(x) \end{aligned}$$

3 $$\begin{aligned}&h_1 = g_1(W_1 {\hat{x}}+b_1)\end{aligned}$$

4 $$\begin{aligned}&{\hat{h}} \sim Dropout(h_1)\end{aligned}$$

5 $$\begin{aligned}&y = g_2(W_2 {\hat{h}}+b_2) \end{aligned}$$

Here, *Dropout*(.) randomly sets a part of input dimension to 0. $g_1(.)$ and $g_2(.)$ are the activation functions for encoder and decoder, respectively. For training the network, the least-square loss, $||x-y||^{2}_{2}$ is minimized. As the activation function, we have used rectified linear units (ReLUs)^51^, for every encoder/decoder pair.

After training, all the encoder and decoder layers are concatenated together, to form a deep autoencoder. The schematic of the deep autoencoder is shown in Figure 3. It is a multilayer deep autoencoder having a bottleneck coding layer in the middle. The reconstructed network is then fine-tuned to minimize the reconstruction loss. We have discarded the decoder layer and used the encoder to generate the initial embedding, $\mathbf {Y_{init}}$.

### Generation of unified probability distribution

For each sample point, *i*, and its potential neighbour, *j*, in the view, *v*, the symmetric probability, $p_{ij}^v$, that *i* selects *j* as its neighbour is given by Eqn 6:

6 $$\begin{aligned} p_{ij}^v= \frac{\exp \{(-d_{ij}^{v})^2\}}{\sum _{k\ne i}\exp \{(-d_{ik}^{v})^2\}} \end{aligned}$$

Here,

7 $$\begin{aligned} p^{v}_{ii} =0 \text { and } \sum _{i,j}p^{v}_{i,j}=1 \end{aligned}$$

The dissimilarity, $d_{ij}^v$ is computed by the Eq. 8. It is the scaled squared Euclidean distance between the high dimensional samples, $x_i$ and $x_j$, in the view *v*.

8 $$\begin{aligned} (d_{ij}^{v})^2 = \frac{||x^{v}_i - x^{v}_j||^2}{2 \{{\sigma ^{v}_i}\}^2} \end{aligned}$$

Here $\sigma ^{v}_{i}$ is generated as such that the entropy of distribution over neighbors equals to $\log k$^52^. Here, *k* is the effective number of nearest neighbors.

After obtaining the Gaussian distribution of each sample point in each view, the combined probability for each sample is generated by conflation method^49^ shown in Eq. 9.

9 $$\begin{aligned} p_{ij} = \frac{\displaystyle \prod _{v} p_{ij}^v}{\displaystyle \prod _{v} p_{ij}^v+\displaystyle \prod _{v}\sum _{k\ne j}p_{ik}^v} \end{aligned}$$

By the basic properties of conflation^53^, the obtained unified probability, $p_{ij}$, is the weighted-squared-mean of the $p_{ij}^v$, and is normal. The obtained *P* is further symmetrized by the Eq. 10.

10 $$\begin{aligned} p_{ij} = \frac{p_{ij} + p_{ji}}{2n} \end{aligned}$$

In the embedded dimension, the induced probability, $q_{ij}$, that $i^{th}$ point will pick $j^{th}$ point as neighbour, is calculated by a Student t-distribution with one degree of freedom^54^, given in Eq. 11. Student t-distribution is used instead of Gaussian because the density of a point is evaluated much faster under Student t-distribution since it does not involve any exponential.

11 $$\begin{aligned} q_{ij} = \frac{\{1+||y_i - y_j||^2\}^{-1}}{\sum _{l\ne k}\{1+||y_l - y_k||^2\}^{-1}} \end{aligned}$$

To find the optimal embedding, the sum of the Kullback-Leibler divergence between the two distributions is minimized as given in Eq. 12:

12 $$\begin{aligned} C = KL(P||Q) = \sum _{i} \sum _{j} p_{ij}\log \bigg (\frac{p_{ij}}{q_{ij}}\bigg ) \end{aligned}$$

#### Extension to incomplete multi-view data-set

In this section, we have shown how our proposed algorithm can be extended for incomplete view settings. In case of incomplete view data, all samples do not appear in every view. The only change that we have to make is in the generation of unified probability, $p_{ij}$. For generating $p_{ij}$ under incomplete view settings, we have used the Eq. 13. When all the views are complete, Eq. 13 is reduced to Eq. 9. From the equation, it can be seen that for the samples occurring in more than one view we have used conflation, but for the points occurring exactly in a single view, we have used the original probability as generated by Eq.6.

13 $$\begin{aligned} p_{ij} = {\left\{ \begin{array}{ll} \quad \frac{p_{ij}^v}{V} &{} \quad \text {when sample }j \text { occurs in only 1 view} \\ &{}\quad \text { but } i \text { occurs in more than 1 view} \\ \quad p_{ij}^v &{} \quad \text {when row }i \text { occurs in only 1 view} \\ \frac{\displaystyle \prod _{v; i,j \in S_{in}} p_{ij}^v}{\displaystyle \prod _{v; i,j \in S_{in}} p_{ij}^v + \displaystyle \prod _{v}\displaystyle \sum _{i,k \in S_{in}; k\ne j}p_{ik}^v} &{} \quad \text {otherwise} \end{array}\right. } \end{aligned}$$

Here, $S_{in}$ is the set of points occurring in more than 1 view.

Rest of the methodologies are similar to those of the complete view setting. Finally, to obtain the optimal embedding, Eq. 12 is minimized.

The generation of unified view is explained with examples in the supplementary file.

### Generation of the final embedding

After obtaining an unified probability distribution ${\mathbf {P}}$ for each sample point in the data set (in Section ) and a combined data set $\mathbf {Y_{init}}$ (in Section ), an unified final embedding ${\mathbf {Y}}$ is generated. At first, instead of randomly initializing ${\mathbf {Y}}$, we initialize it with $\mathbf {Y_{init}}$. We start by calculating $q_{ij}$ using Eq. 11 for ${\mathbf {Y}}$, and try to minimize the KL-divergence in Eq. 12. Using stochastic gradient descent the values of *Y* are optimized. Finally, the embedding ${\mathbf {Y}}$ is obtained. The working methodology of the proposed algorithm is shown in Algorithms 1 and 2.

### Optimization

The KL divergence between the two joint probability distributions, *P* and *Q*, is given by Eq. 12. The equation can be written as:

14 $$\begin{aligned} C = \sum _i \sum _j p_{ij}\log {p_{ij}}- p_{ij}\log {q_{ij}} \end{aligned}$$

Before performing the derivation, we define the following terms

15 $$\begin{aligned} {\overline{d}}_{ij}= & {} ||y_i-y_j|| \end{aligned}$$

16 $$\begin{aligned} S= & {} \sum _{k \ne l} \{1+{\overline{d}}_{lk}^2\}^{-1} \end{aligned}$$

Now, for change in $y_i$, the pairwise distances that may change are ${\overline{d}}_{ij}$ and ${\overline{d}}_{ji}$, $\forall j$. Hence, the gradient of the cost function, *C*, with respect to $y_i$ is given by:

17 $$\begin{aligned} \frac{\partial C}{\partial y_i}&= \sum _j \bigg (\frac{\partial C}{\partial {\overline{d}}_{ij}} + \frac{\partial C}{\partial {\overline{d}}_{ji}}\bigg ) (y_i - y_j) \end{aligned}$$

18 $$\begin{aligned}&= 2 \sum _{j}\frac{\partial C}{\partial {\overline{d}}_{ij}}(y_i - y_j) \end{aligned}$$

$\frac{\partial C}{\partial {\overline{d}}_{ij}}$ is obtained by differentiating KL-divergence in Eq. 14.

$$\begin{aligned} \frac{\partial C}{\partial {\overline{d}}_{ij}}&= - \sum _{k \ne l} p_{kl} \frac{\partial \log q_{kl}}{\partial {\overline{d}}_{ij}} \\&= -\sum _{k \ne l} p_{kl}\bigg (\frac{1}{q_{kl}S}\frac{\partial ((i + {\overline{d}}_{kl}^2)^{-1})}{\partial {\overline{d}}_{ij}}-\frac{1}{S}\frac{\partial S}{\partial {\overline{d}}_{ij}}\bigg ) \end{aligned}$$

For k = i and l = j, the gradient $\frac{\partial ((i + {\overline{d}}_{kl}^2)^{-1})}{\partial {\overline{d}}_{ij}}$ is non zero and also $\sum _{k \ne l} p_{kl} =1$. Hence, the gradient, $\frac{\partial C}{\partial {\overline{d}}_{ij}}$, is given by,

19 $$\begin{aligned} \frac{\partial C}{\partial {\overline{d}}_{ij}} = 2(p_{ij}-q_{ij})(1+{\overline{d}}_{ij}^2)^{-1} \end{aligned}$$

Substituting this in Eq. 18, we have the final gradient as:

20 $$\begin{aligned} \frac{\partial C}{\partial y_{i}}= 4 \sum _{j}(p_{ij}-q_{ij})(1+||y_i-y_j||^2)^{-1}(y_{i}-y_{j}) \end{aligned}$$

### Generation of clusters

Here, we have discussed about the algorithm used to generate the clusters from the embedded sample points.

#### Multi-objective clustering technique

Multi-objective optimization (MOO) based clustering algorithms are better in capturing clusters having different shapes^55^ and can detect the number of clusters automatically from the data set. For our experiment, we have used the algorithm, Archived Multi-objective Simulated Annealing (AMOSA), similar to that used in^56^. We have used center based encoding. The centers of the clusters are encoded in a solution to represent a partitioning. The concepts of variable length encoding are used to automatically identify the number of clusters from a data set. The number of clusters in different solutions are varied over a range. The choice of the algorithm is not restricted to this, any other MOO based algorithm can be used.

- *Objective functions used* For our experiments, we have simultaneously optimized two objective functions, viz., Xie-Beni(XB) index^57^ and PBM index^58^.
  **XB-index**^57^ computes the ratio between the cluster compactness and cluster separation. The minimum value of XB-index represents the optimal partitioning.
  21 $$\begin{aligned} XB = \frac{\sum _{q=1}^{K} \sum _{r=1}^{n} \mu _{qr}^{2}d(\overline{x}_{r} , \overline{C}_{q})}{n(min_{l\ne m} d(\overline{C}_{l},\overline{C}_{m}))}, \end{aligned}$$
  where *K* = number of clusters
  $$\begin{aligned} \mu _{qr} = \left\{ \begin{array}{lr} 1 &{} : r^{th}\text{-data } \text{ point } \in q^{th}\text{-cluster }\\ 0 &{} : \text{ otherwise, } \end{array} \right. \end{aligned}$$
  $d(\overline{x}_{r} , \overline{C}_{q})$ = distance between the cluster center and the points within the cluster, $d(\overline{C}_{l},\overline{C}_{m}))$ = distance between cluster centers.
  **PBM index**^58^ is defined as follows:
  22 $$\begin{aligned} PBM(K)=\left( \frac{1}{K}\times \frac{{\mathscr {E}}_1}{\mathscr {E_K}}\times D_K\right) \end{aligned}$$
  Here, *K* = number of clusters, $D_K=\max _{k,l=1}^{K}d(\overline{C}_k,\overline{C}_l)$ and $\mathscr {E_K}= \sum _{k=1}^{K}\sum _{l=1}^{n_k}d(\overline{x}_l^k,\overline{C}_k)$. Here, $\overline{x}_l^k$ = $l^{th}$ point of the $k^{th}$ cluster, $\overline{C}_k$ = the center of the $k^{th}$ cluster and $n_k$ = samples in the $k^{th}$ cluster. Maximum value of PBM index corresponds to the optimal number of clusters.
- *Mutation operator* There are three mutation operators to explore the search space:
  - *Normal mutation* Under this technique, a cluster center is randomly selected and its feature values are replaced by random values drawn from the Laplacian distribution. The distribution is given by, $p(\epsilon )\propto e^{-\frac{|\epsilon -\mu |}{\delta }}$ with $\mu$= old value at the cluster center and $\delta$= 1.0 (sets the magnitude of perturbation).
  - *Insert mutation* Under this case, a set of solutions are randomly selected and the corresponding number of clusters are increased by 1.
  - *Delete mutation* Under this case, a set of solutions are randomly selected and the corresponding number of clusters are decreased by 1.

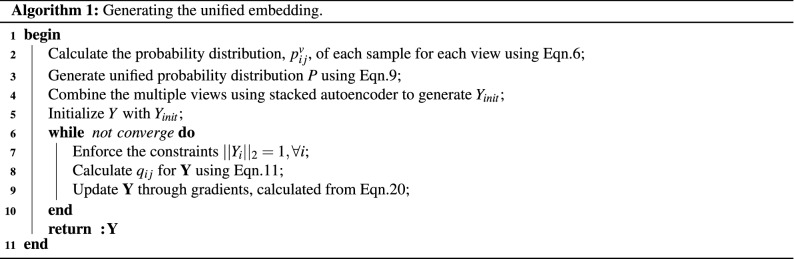

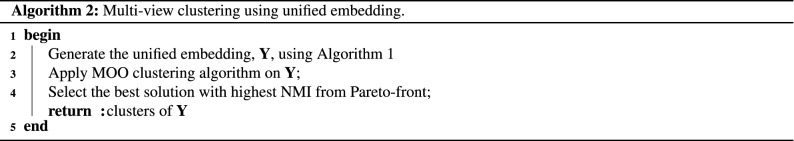

## Results

This section gives an overview of the datasets used in our experiment, experimental setup and some of the results obtained.

### Data sets

For performance evaluation of *MvNE*, experiments are performed on 10 benchmark omics data sets downloaded from https://tcga-data.nci.nih.gov/tcga/. For all the data sets, following three views are used: gene expression, miRNA expression and DNA Methylation. Specifications of the datasets are shown in Table 1. Clinical data is used for obtaining the clusters from each dataset. Brief descriptions of the datasets are given below:

**Table 1:** Description of the different views of the data sets. The numbers in the brackets are selected features.

Dataset	No. of features			Samples	#Clusters
	Gene expression	miRNA expression	DNA methylation		
*BRCA*	20510 (400)	1046 (220)	4885 (400)	684	4
*GBM*	12042 (400)	534 (110)	5000 (400)	274	4
*OVG*	12043 (400)	800 (190)	5000 (400)	291	3
*COAD*	20351 (400)	705 (170)	5000 (400)	221	4
*LIHC*	20531 (400)	705 (170)	5000 (400)	410	4
*LUSC*	20531 (400)	705 (170)	5000 (400)	344	4
*SKCM*	20531 (400)	705 (170)	5000 (400)	450	4
*SARC*	20531 (400)	1046 (241)	5000 (400)	217	4
*KIRC*	20531 (400)	705 (170)	5000 (400)	212	4
*AML*	20531 (400)	705 (168)	5000 (400)	170	2

#### Breast cancer (BRCA)

This Breast cancer dataset contains samples from patients. The breast cancer dataset have 4 clusters: Her2, Basal,LumA, LumB^59,60^.

#### Glioblastoma multiforme (GBM)

This dataset cotai patient samples suffering from GBM. GBM can be classified into four different types^61^: Classical, Mesenchymal, Neural and Proneural.

#### Ovarian cancer (OVG)

Details of the patients having ovarian serous cystadenocarcinoma tumors are listed in this dataset. Based on the cancer stages thre are three groups: stage II/A/B/C ; III/A/B/C and IV.

#### Colon cancer (COAD)

Data from patients suffering from Colon Adenocarcinoma are present here. Based on different stages of the cancer it is divided into 4 groups: stage I/A/B/C; II/A/B/C; III/A/B/C and IV/A/B.

#### Liver hepatocellular carcinoma(LIHC)

Data from patients suffering from liver cancer are listed here. Based on different stages of the cancer it is divided into 4 groups: I; II; III/A/B/C; and IV.

#### Lung squamous cell carcinoma (LUSC)

Data from patients suffering from lung cancer are present here. Based on different stages of the cancer it is divided into 4 groups: I/A/B/C; II/A/B; III; and IV.

#### Skin cutaneous melanoma(SKCM)

Data from patients suffering from Melanoma cancer is present in the dataset. Based on different stages of the cancer it is divided into 4 groups: 0, II/A/B/C; III/A/B/C; and IV/A/B/C.

#### Sarcoma (SARC)

SARC contains samples from patients suffering from sarcoma. Based on the cancer types it has four main groups: Leiomyosarcoma, Dedifferentiated liposarcoma, Pleomorphic MFH and Myxofibrosarcoma.

#### Kidney renal clear cell carcinoma (KIRC)

KIRC contains samples from patients suffering from kidney cancer. Based on different stages of the cancer it is divided into 4 groups: I/A/B/C, II/A/B/C; III/A/B/C; and IV/A/B/C..

#### Acute myeloid leukemia (AML)

AML dataset contains samples from patients suffering from Leukemia. Based on the type of cancer it is divided into 2 groups: Qiet and Kirc+.

### Comparing methods

Under this section, we have discussed about the algorithms that were used for comparison. Two baseline methods are developed based on the different techniques for combining probabilities. The details are as follows:

1. *MCCA*^62^: Canonical correlation analysis (CCA) works with only two views. Witten and Tibshirani proposed sparse multiple CCA (MCCA) which supports more than two views. It operates by maximizing the pairwise correlation between projections and CCA-RLS^63^.
2. *MultiNMF*^11^: It performs NMF on each view individually: Each omic $D^v$ is factorized into $W^vH^v$. The omics are then integrated by enforcing the constraint that the $W^v$ matrices are close to the “concensus” matrix *W*.
3. *DiMSC*^32^: Diversity-induced Multi-view Subspace Clustering (DiMSC) uses Hilbert Schmidt Independence Criterion (HSIC) as diversity term to exploit the complementary information between different views.
4. *LRACluster*^64^: This uses a latent sample representation to determine the distribution of the features. It optimizes a convex objective and offers a solution that is globally optimal.
5. *PINS*^47^: To combine clusters of different views, it uses a connectivity matrix. The number of the clusters is chosen in such a way that the perturbation is robust. Perturbation is obtained by adding Gaussian noise to the data.
6. *SNF*^41^: It is a similarity-based approach that generates a similarity network separately for each view. Such networks are fused together by an iterative process.
7. *iClusterBayes*^65^: It uses Bayesian regularization based joint latent-variable model to detect the clusters from multi-omics data.
8. *MVDA*^44^: In this approach, the information from various data layers (views) is incorporated at the result stage of each single view clustering iteration. This functions by factorizing the membership matrices in a late integration manner.
9. *MvNE:* Our proposed multi-view clustering methodology uses conflation method to combine the views in the probabilistic domain and generates an unified embedding. We have applied multi-objective optimization algorithm, AMOSA^56^, on the embedded data sets to obtain the clusters. AMOSA automatically determines the number of clusters from the data set. From the obtained Pareto-front, we have reported the results of the solutions which have high NMI values.
10. *AvgProb:* As a baseline method, we have at first generated a probability distribution of the samples on each view and then combined the distributions by considering the average of the probabilities over the views. Final embedding is generated by minimizing the KL divergence between the obtained average probability and the probability in embedded domain.
11. *AvgData:* As a baseline method, we have generated a combined distance matrix by considering the average of distance matrices from all the three views. This probability distribution in the higher dimension is generated from this combined distance matrix. Final embedding is generated by minimizing the KL divergence between the generated probability and the probability in the embedded domain.

### Preprocessing of data sets

Most omics data sets have a much smaller number of samples than the number of features. To manage different distributions, feature normalization in different omics data is important. In addition, dimensionality reduction/ feature selection is necessary to provide equal opportunities to different omics data in clustering process. Reduction of dimensionality is also important for retaining the most significant features, reducing computation load. We have used an unsupervised technique for choice of features, variance ranking, in our approach. We have measured the variance of each feature for this. For gene expression and DNA methylation data sets, top 400 features with highest variance scores are selected. For miRNA sequence, top 22–24% features are selected. This is because miRNA sequences have less number of features compared to other two.

### Experimental settings

For state-of-the-art methods, we have used the codes released by corresponding authors. For our method, empirically we have selected the size of low dimensional embedding, *dim*, as 80 for all the data sets. The size of the nearest neighbour, *k*, is set to 30 empirically for all data sets. The total iteration of gradient descent is set to 2000, initial momentum is set to 0.5 and final momentum is set to 0.9. Initially learning rate ($\mathbf {\eta }$) is set to 200, and after every iteration, it is updated by adaptive learning rate scheme described by Jacobs et al.^66^.

### Evaluation metrics

Two measurement indices, *normalized mutual information* (NMI)^67^ and *adjusted rand index* (ARI)^68^ are used to compare MvNE with other approaches. Both metrics measure the differences between the real and the predicted partitions; higher values indicate more similarity with the predicted group.

23 $$\begin{aligned} NMI(C,E) = \frac{2\times I(C;E)}{H(C) + H(E)} \end{aligned}$$

Here *C* and *E* are the true class labels and cluster labels, respectively. *I*(.) is the mutual information, *H*(.) is the entropy.

### Parameters study

**Figure 4:**
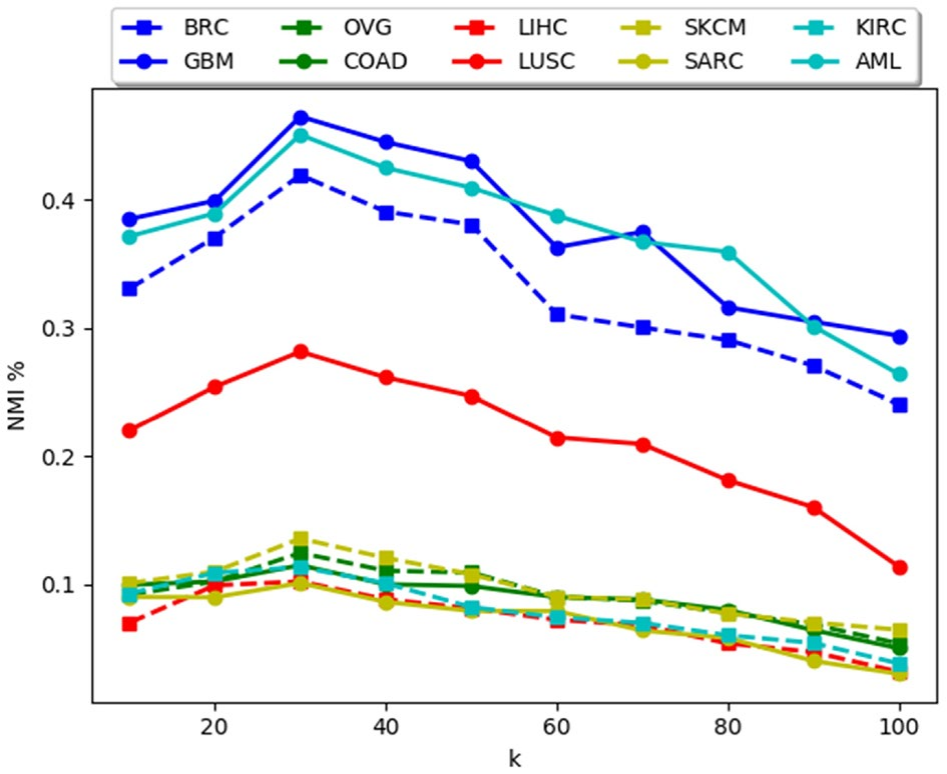
Change in NMI(%) with changes in k.

**Figure 5:**
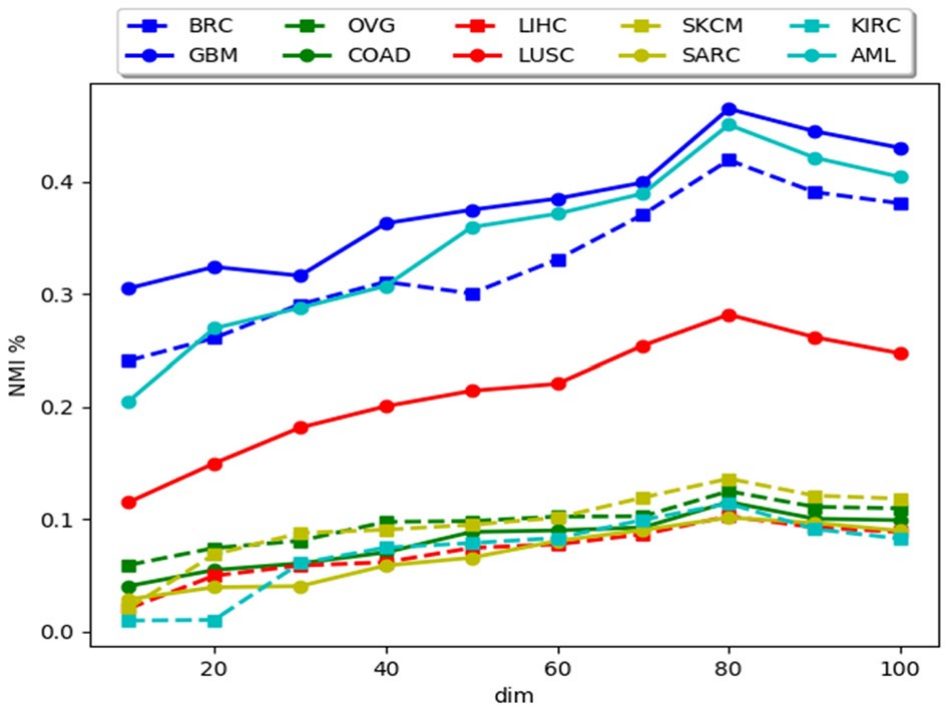
Change in NMI(%) with the changing dimension (*dim*) of the embedded dataset.

There are two main parameters in the proposed methodology, i.e., the size of the *k* nearest neighbors (*k*) and the unified embedding dimension (*dim*). Under this section, we have analyzed the performance of *MvNE* with changes in these parameters. Results on all the ten data sets are reported in Figs. 4 and 5.

From Fig. 4 it is evident that, when *k* is too small, the probability distribution has very little information regarding the global structure of the clusters and it is too much focused on the local structure which causes the clusters to break into several sub clusters deteriorating the performance of the algorithm. If the *k* is too large, it fails to capture the structures of the clusters properly, causing merging of clusters and the algorithm is not stable when *k* is large. Empirically we have set the value of *k* to 30 for all data sets.

Figure 5 shows that, when *dim* is too small, unified embedding fails to capture enough information to reflect the structure of the data set. When it is too large, the performance degrades. One of the reasons for this is the use of Student-t distribution with one degree of freedom. With the increase in dimension it fails to preserve the local structure of the data set because in higher dimension, the heavy tails comprise of a relatively large portion of the probability mass. Empirically we have kept the embedded dimension to 80 for all data sets.

In SAE, we have kept the input layer size to the size of the input vectors, for example, like for gene expression values, we have kept input dimension size to 400. There are three hidden layers of size, 500, 80 and 500, respectively. The output layer has same size that of the input layer. The dropout value is set to $5\%$.

For the multi-objective clustering algorithm, we have set the parameters in accordance to^56^. We did not tune the settings of AMOSA^69^ because the main focus of the paper is on generating the optimal embedding. The parameter settings are as follows: $T_{max}=100$, $T_{min}=0.001$, $Iteration=100$, *rate of cooling*$=0.9$, *Min clusters*$=2$, *Max clusters*$=\root \of {\{samples\}}$, $SL=50$ and $HL=40$. The algorithm is executed for 20 times.

### Gene marker identification

BRCA data set includes four groups of patients, i.e., LumB, LumA, Her2 and Basal. A binary classification problem is solved to identify the most significant genes from each class. Two groups, one with samples from one class and the other with samples from other classes are formed. Signal-to-noise ratio (SNR)^70^ is determined for each gene after considering both classes. It is described as,

24 $$\begin{aligned} SNR = \frac{\mu _{1}-\mu _{2}}{\sigma _{1}+\sigma _{2}} \times 100, \end{aligned}$$

Here $\mu$ is the mean and $\sigma$ is the standard deviation of each class.

Genes with high SNR values correspond to high value of expression for the class to which they belong and vice versa. Finally, 5 up regulated (high SNR) and 5 down regulated (lowest SNR values) genes are selected from SNR list.

Table 2 shows the list of selected gene markers for all the classes.

**Table 2:** 5 up regulated and 5 down regulated Gene markers for BRCA dataset.

LumA	LumB	Her2	Basal
** Up regulated genes**			
CIRBP	ARL6IP1	ERBB2	DSC2
TENC1	PTGES3	GGCT	YBX1
KIF13B	PCNA	ACTR3	FOXC1
COL14A1	PLEKHF2	STARD3	ANP32E
NTN4	SFRS1	GRB7	PAPSS1
** Down regulated genes**			
TUBA1C	TRIM29	GREB1	XBP1
FOXM1	PPL	ESR1	GATA3
MYBL2	SFRP1	MAPT	ZNF552
MKI67	ZFP36L2	TBC1D9	MLPH
TPX2	NDRG2	BCL2	FOXA1

**Figure 6:**
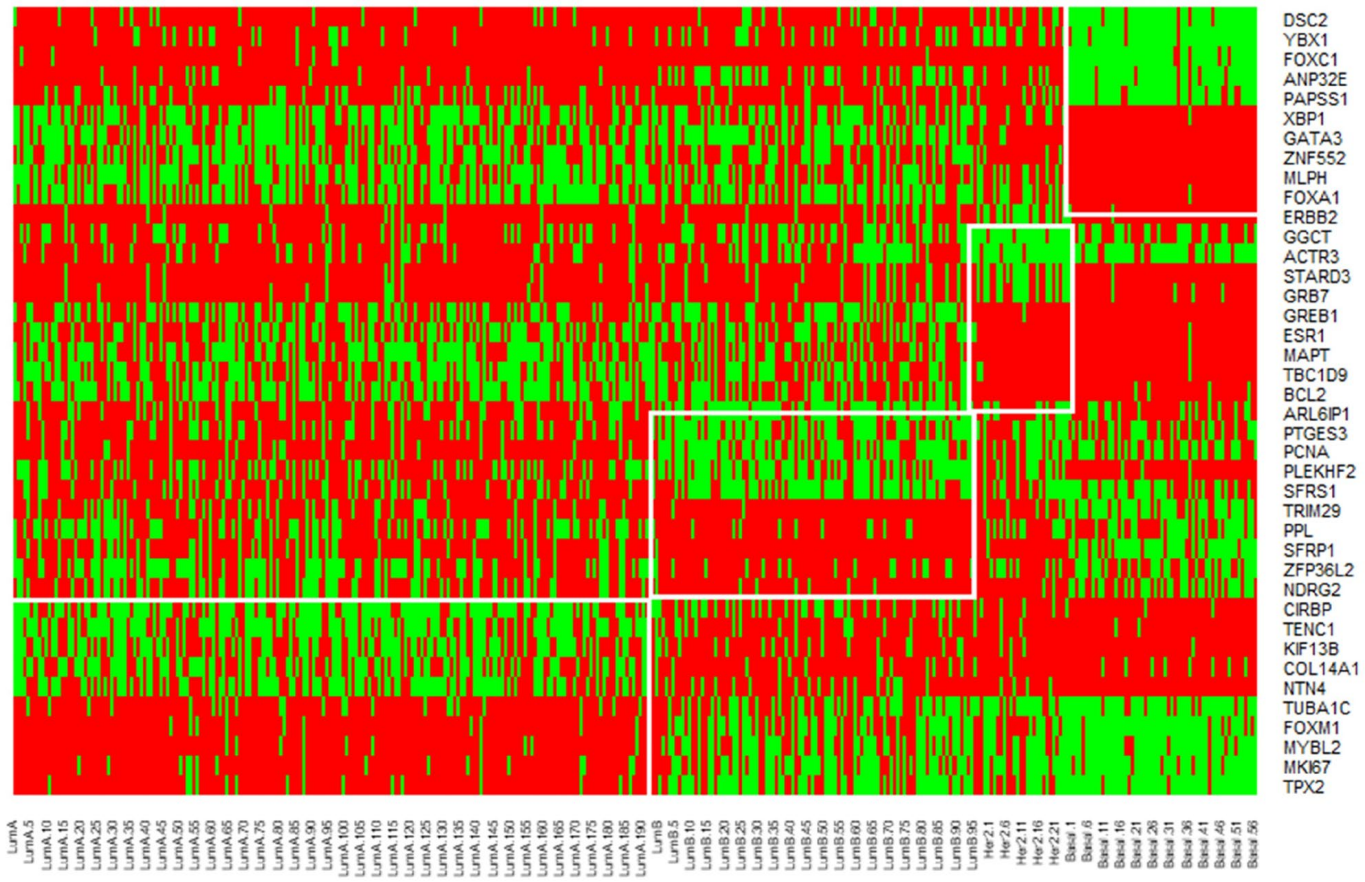
Heatmap showing the levels of expression of selected gene markers in the BRCA dataset for each subclass.

**Figure 7:**
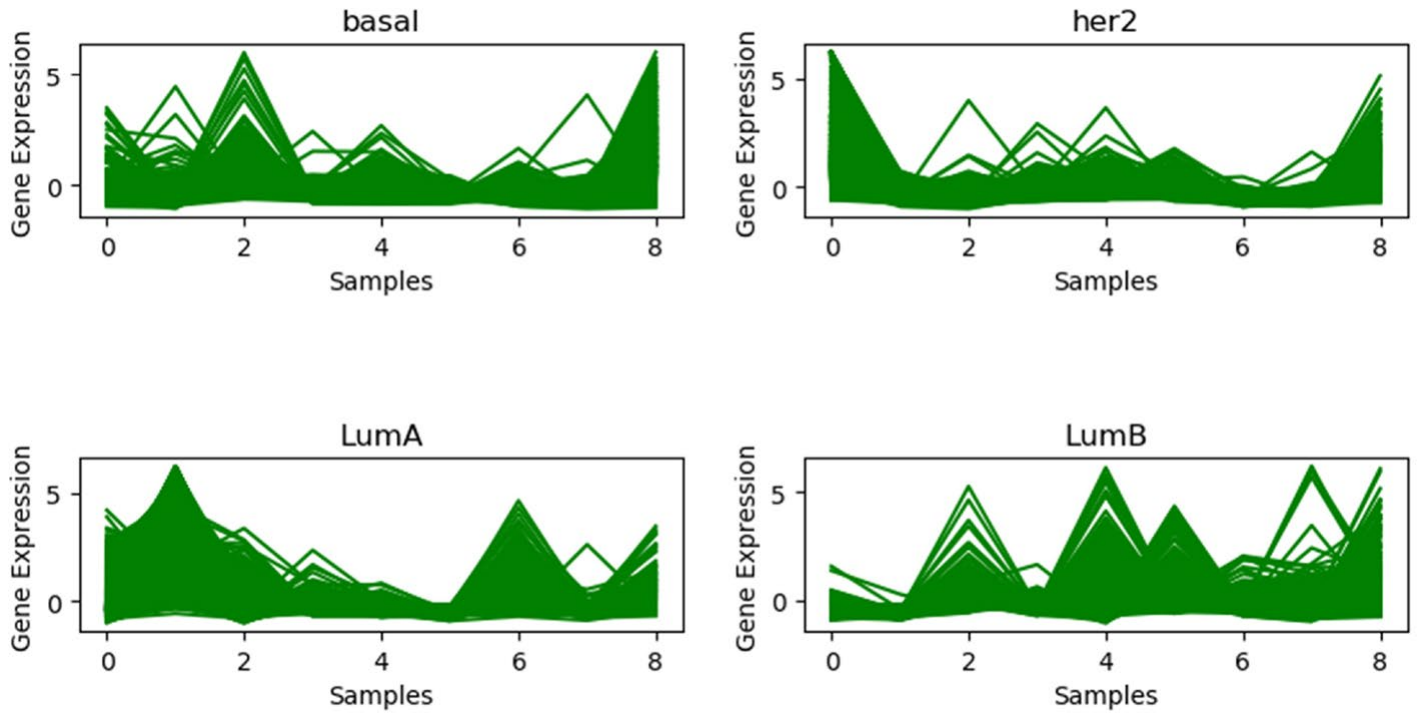
Gene expression profile plot in the BRCA dataset for each subclass.

**Table 3:** The p-values obtained on comparing *MvNE* with other comparing methods in terms of NMI.

	BRCA	GBM	OVG	COAD	LIHC	LUSC	SKCM	SARC	KIRC	AML
MCCA	0.0014	0.0091	0.0031	0.0184	0.0151	0.0073	0.0043	0.0269	0.0302	0.02546
MultiNMF	0.0086	0.0076	0.0051	0.0303	0.0054	0.0019	0.0089	0.0214	0.0275	0.0139
DiMSC	0.0056	0.0034	0.0051	0.0291	0.0104	0.0078	0.0035	0.0235	0.0207	0.0201
LRAcluster	0.0036	0.0211	0.0215	0.0012	0.0036	0.0051	0.0062	0.0084	0.0051	0.0167
PINS	0.0044	0.0112	0.0008	0.0273	0.0089	0.0045	0.0244	0.0062	0.0065	0.0163
SNF	0.0126	0.0057	0.0086	0.0076	0.0277	0.0062	0.0119	0.00634	0.0026	0.0062
iClusterBayes	0.0045	0.0118	0.0086	0.0124	0.0357	0.0023	0.0076	0.0048	0.0034	0.0043
MVDA	0.0042	0.0132	0.0071	0.0051	0.0043	0.0073	0.0009	0.0015	0.0021	0.0077
AvgProb	0.0051	0.0178	0.0012	0.0012	0.0057	0.0031	0.0064	0.0068	0.0074	0.0053
AvgData	0.0061	0.0113	0.0091	0.0051	0.0049	0.0028	0.0098	0.0041	0.0092	0.0062

### Statistical significance test

The significance test is carried out using one-way Analysis of Variance (ANOVA) test at $5\%$ significance level. The results obtained by our proposed methodology *MvNE* over 20 runs are compared with other algorithms. The p-values are shown in the Table 3. The reported p-values indicate that statistically significant results are obtained.

## Discussion

In Tables 4 and 5, we have compared the NMI and Adjusted Rand Index (ARI) values over 10 omics data sets obtained by different clustering methods. In terms of NMI and ARI, our proposed methodology, *MvNE*, shows an improvement of 2–3% and 1.8–2.9% over all the data sets with respect to state-of-the-art algorithms, respectively. The maximum NMI and ARI values are marked in bold in Tables 4 and 5 respectively.

From the results, it can be seen that iClusterBayes^65^ performs poorly as it may get stuck at local optimal solutions due to complex structure.

Further in Table 6, we have also reported the $macro\text { }F1-score$ and *Accuracy* results obtained by our proposed methodology for all the 10 omic datasets.

The baseline method, *AvgProb*, performs poorly as average of the probabilities failed to capture the structure of the probability distribution as discussed under the “Methods”. *MvNE* outperforms both the baseline methods, *AvgData* and *AvgProb*, showing the superiority of the conflation method.

Our hypothesis of generating subspaces by combining different views in the probabilistic domain proves effective with the results obtained.

The BRCA dataset has 4 classes, so a total of 40 genes with 20 down-regulated genes and 20 up-regulated are obtained. Fig 6 shows the heatmap plot of these genes. Here, red means higher levels of expression values, green means lower levels of expression, and black means moderate levels of expression. Fig 6 also indicates that genes known for a specific tumor class are either down-regulated or up-regulated.

We have listed the gene expression profile plot for each BRCA dataset group in Fig 7. The structure compactness shows that the clustered samples have the same form of gene expression, i.e., there is a strong continuity between them within a cluster sequence.

In Table 3, we have reported the p-values obtained by our proposed model when compared with other state-of-the-art and baseline models. These results are below $5\%$ significance level. This shows that performance improvements obtained by our proposed model, *MvNE*, are statistically significant.

**Table 4:** Comparison results in terms of NMI.

	BRCA	GBM	OVG	COAD	LIHC	LUSC	SKCM	SARC	KIRC	AML
MvNE	**0.4192**($\pm 0.15$)	**0.4449**($\pm 0.18$)	**0.1247**($\pm 0.01$)	**0.1151**($\pm 0.02$)	**0.1024**($\pm 0.02$)	**0.2816**($\pm 0.02$)	**0.1359**($\pm 0.01$)	**0.1512** ($\pm 0.01$)	**0.1137**($\pm 0.01$)	**0.4507**($\pm 0.11$)
MCCA	0.2086	0.2865	0.0731	0.0784	0.0546	0.2031	0.0952	0.0993	0.0765	0.3046
MultiNMF	0.3001	0.3606	0.0713	0.0657	0.0489	0.2401	0.0981	0.0836	0.0755	0.2787
DiMSC	0.3856	0.4089	0.1015	0.0917	0.0806	0.2598	0.0996	0.1051	0.0892	0.3166
LRAcluster	0.0146	0.0532	0.0304	0.0328	0.0573	0.0672	0.0483	0.0475	0.0389	0.3629
PINS	0.0118	0.0153	0.0095	0.0459	0.0348	0.0237	0.0382	0.0262	0.0279	0.2219
SNF	0.3581	0.026	0.0068	0.0332	0.0129	0.0082	0.0088	0.0233	0.0908	0.4349
iClusterBayes	0.0121	0.0306	0.0081	0.0106	0.0258	0.0112	0.0044	0.0177	0.0108	0.0894
MVDA	0.3912	0.4213	0.1063	0.0993	0.0775	0.2694	0.1195	0.1121	0.0955	0.2871
AvgProb	0.0119	0.0281	0.0092	0.0151	0.0261	0.0108	0.0038	0.0107	0.0109	0.0604
AvgData	0.3804	0.4053	0.1035	0.09193	0.0705	0.2644	0.1007	0.0891	0.1008	0.3014

**Table 5:** Comparison results in terms of ARI.

	BRCA	GBM	OVG	COAD	LIHC	LUSC	SKCM	SARC	KIRC	AML
MvNE	**0.2623**($\pm 0.02$)	**0.3609**($\pm 0.013$)	**0.0957**($\pm 0.01$)	**0.0561**($\pm 0.01$)	**0.0215**($\pm 0.004$)	**0.1571**($\pm 0.03$)	**0.0560**($\pm 0.02$)	**0.0715**($\pm 0.01$)	**0.0937**($\pm 0.01$)	**0.3915**($\pm 0.14$)
MCCA	0.1903	0.2243	0.03031	0.0182	0.0031	0.1012	0.0093	0.0188	0.0195	0.1846
MultiNMF	0.2107	0.25476	0.04112	0.0163	0.0041	0.1107	0.0102	0.0208	0.0203	0.1964
DiMSC	0.2189	0.2806	0.0503	0.0191	0.0061	0.1142	0.0113	0.0212	0.0341	0.2137
LRAcluster	0.0086	0.0076	0.0051	0.0184	0.0054	0.0098	0.0055	0.0263	0.0392	0.2546
PINS	0.0144	0.0089	0.0045	0.0244	0.0067	0.0065	0.0016	0.0152	0.0136	0.1195
SNF	0.0126	0.0027	0.0062	0.0119	0.0063	0.0026	0.00062	0.0238	0.0157	0.3667
iClusterBayes	0.0045	0.0357	0.0023	0.0076	0.0048	0.0034	0.0043	0.0399	0.0288	0.0482
MVDA	0.2457	0.3441	0.0614	0.0194	0.0085	0.1351	0.0145	0.02366	0.0355	0.2021
AvgProb	0.021	0.0512	0.0091	0.00651	0.0096	0.0851	0.0013	0.0209	0.0205	0.0508
AvgData	0.2107	0.3404	0.0716	0.1941	0.0091	0.1261	0.01321	0.01326	0.01201	0.2709

**Table 6:** macro F1-score and Accuracy values obtained by *MvNE* for all the datasets.

Datasets	macro F1-score	Accuracy
BRCA	0.6632	0.6701
GBM	0.6801	0.6918
OVG	0.4883	0.4765
COAD	0.4514	0.4531
LIHC	0.4457	0.4612
LUSC	0.5856	0.5771
SKCM	0.5031	0.5118
SARC	0.5503	0.5517
KIRC	0.4675	0.4718
AML	0.6904	0.7013

### Theoretical analysis

#### Time complexity

The proposed methodology can be divided into three parts, viz., generation of the initial embedding using SAE, generation of the final low dimensional embedding and finally the AMOSA algorithm for clustering. The time complexity of each part of the algorithm is as follows:

1. *Time complexity of SAE* In our proposed approach, we have used 3 hidden layers. The time complexity of matrix multiplication is $$M_{ij} * M_{jk}$$ is $$O(j*j*k)$$.
  Since, we have total 4 layers (3 hidden layers and 1 output layer), so we require total 4 weight matrices to compute the output layer, say, $$W_{ji}, W_{kj}, W_{lk}$$ and $$W_{ml}$$. For a training sample *t*, number of iterations as *n* and input vector of size, *i*; the time complexity for total forward and backward pass is typically: $$O(n*t*(ij+jk+kl+lm))$$
  However, we have parallelized the SAE by using GPUs.
2. *Generation of embedding from high dimensional space* The time complexity of generating the low dimensional embedding for *N* number of samples is $O(N*N)$. So, for very large dataset, this method is very slow. In the supplementary file, we have shown results on large datasets having more than 3000 samples. But this algorithm is best suited for biological datasets where less number of samples are available.
3. *Time complexity of AMOSA* For a population size of *P*, iteration of *iter* and *N* number of samples, AMOSA has time complexity of $O(NlogN * P * iter)$

#### Convergence analysis

**Figure 8:**
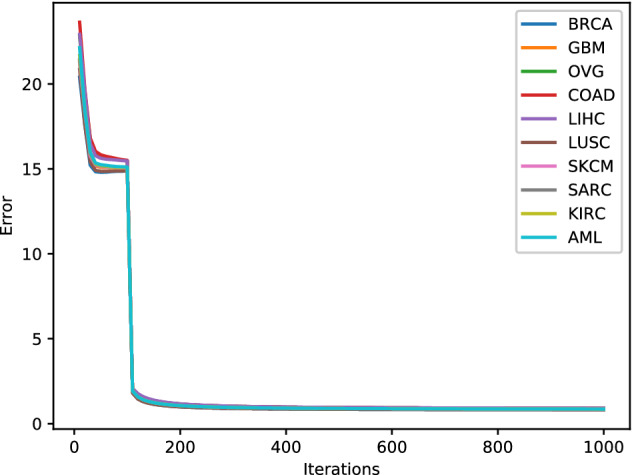
Error plot for low dimension generation.

For convergence analysis of our algorithm, we have shown the error plots for all the datasets while generating the low dimensional embedding. From Fig. 8, it can be observed that for 1000 iterations, there is a monotonic decrease in the error value for all the datasets. This shows the convergence of our proposed methodology.

$$O(n*t*(ij+jk+kl+lm))$$

## Conclusion

In this paper, we have proposed an unsupervised probabilistic approach to generate an unified neighbourhood embedding for multi-view data sets. The proposed methodology combines the multiple omics data in the probability space and then generates an unified embedding preserving the statistical properties of each view as well as the combined neighbourhood information of the samples. Assigning equal weightage to each view is not very likely for solving patient classification problems. One of the key benefits of our proposed methodology is that it utilizes a weighted combinations of views. The conflation method used here for combining different omics data, automatically assigns high weightage to the more accurate omics data. Another advantage of the proposed methodology is that it can handle data having incomplete views, i.e., missing samples in some views. The results for incomplete views are shown in the supplementary file. However, one of the major drawbacks of the proposed methodology is the time complexity of calculating the embedding in the lower dimensions. It has very high time complexity. So for large datasets, the algorithm is slow. This algorithm is best suited for medium sized datasets, like patient stratification datasets where the number of samples are generally low. Results on 10 omics datasets illustrate that our methodology provides better results.

## Supplementary information

Supplementary information.
