# Supplementary material for: Multi-view clustering for multi-omics data using unified embedding
Source: Sci Rep. 2020 Aug 12;10:13654. doi: 10.1038/s41598-020-70229-1 (PMC7423957; doi:10.1038/s41598-020-70229-1)
Supplement: Supplementary file 1 — Supplementary information. [file 41598_2020_70229_MOESM1_ESM.pdf]

Supplementary: Multi-view Clustering for Multi-Omics Data Using Unified Embedding

Sayantan Mitra[1,*], **Sriparna Saha**[1], **and Mohammed Hasanuzzaman**[2]

[1]Department of Computer Science, Indian Institute of Technology Patna, Bihta, Bihar, 801103, India
[2]ADAPT Centre, Cork Institute of Technology, Ireland
[*]sayantaniem@gmil.com

Example of the conflation of Probability

Suppose, A_v is the vth view of a dataset A. The A_v matrix is shown below:

$$A_v = \begin{pmatrix} 0.56950374 & 0.24939832 & 0.59730345 & 0.97707559 & 0.80886214 & 0.49921964 & 0.62260146 & 0.53265819 \\ 0.52036463 & 0.46893455 & 0.06359224 & 0.05452818 & 0.87890188 & 0.14043775 & 0.69887107 & 0.38344646 \\ 0.4463943 & 0.00589088 & 0.05203859 & 0.46290895 & 0.23644766 & 0.30070013 & 0.31682137 & 0.04161984 \\ 0.5360699 & 0.08522395 & 0.46348496 & 0.32214276 & 0.67780069 & 0.01471646 & 0.59955487 & 0.37604494 \\ 0.67314118 & 0.24470446 & 0.93019923 & 0.71523027 & 0.92886573 & 0.3667204 & 0.92863226 & 0.4496676 \end{pmatrix}$$

Each row of A_v represents a sample and the columns represent its corresponding features. A_v matrix has 5 samples, each represented by a 8 dimensional vector. Now, we generated a probability matrix P from this matrix A_v. The P matrix is shown below:

$$P = \begin{pmatrix} 0. & 0.08686422 & 0.08853805 & 0.25837206 & 0.56622567 \\ 0.11142199 & 0. & 0.18107563 & 0.60091382 & 0.10658856 \\ 0.15250879 & 0.24756775 & 0. & 0.55498816 & 0.04493529 \\ 0.1039803 & 0.60448491 & 0.16465764 & 0. & 0.12687714 \\ 0.53132751 & 0.11750749 & 0.04988539 & 0.3012796 & 0. \end{pmatrix}$$

P is a square matrix of size $sample \times sample$. Here, the size of P is 5×5. Now, P_{ij} represents what is the probability that j^{th} point is the neighbourhood of ith point.The summation of each row of matrix P is 1.

Similarly, for each view the probability matrix is generated. The next task is to combine these probability matrix to generate a unified probability matrix P_{uni}. From the P_{uni} the low dimension embedding is generated.

There are two possible scenarios, (i) All the views are complete and (ii) Views are incomplete. For showing the conflation method, we have shown the example with a single row matrix and the same process is repeated for each row of the matrix.

For Complete View

Let us assume we have two views, V1 and V2, and the probability distributions of the views are as follows:

$$V_1 = \begin{pmatrix} 0.6 & 0.2 & 0.2 \end{pmatrix}$$
$$V_2 = \begin{pmatrix} 0.7 & 0.2 & 0.1 \end{pmatrix}$$

This distribution is combined as follows:

$$V_{uni} = \begin{pmatrix} \frac{0.6\times0.7}{0.6\times0.7+(0.2+0.2)\times(0.2+0.1)} & \frac{0.2\times0.2}{0.2\times0.2+(0.6+0.2)\times(0.7+0.1)} & \frac{0.2\times0.1}{0.2\times0.1+(0.6+0.2)\times(0.7+0.2)} \end{pmatrix}$$
$$= \begin{pmatrix} 0.77 & 0.058 & 0.05 \end{pmatrix}$$

Now, to make the summation of all the probabilities= 1, we do the following adjustments:

$$\frac{1-(0.77+0.058+0.05)}{3} = 0.041(approx)$$
$$V_{uni} = \begin{pmatrix}0.77+0.041 & 0.058+0.041 & 0.05+0.041\end{pmatrix} = \begin{pmatrix}0.811 & 0.099 & 0.091\end{pmatrix}$$

For any number of views, the same procedure is followed.

For Incomplete View

Under incomplete view settings, there can be two scenarios (i) a sample is present in a single view (ii) the sample is present in more than 1 view but not in all the views (this case occurs for more than 2 view scenario).

2 View case

At first, we have shown the results for two view scenarios. The probability distributions of the views, V_1 and V_2, are as follows:

$$V_1 = \begin{pmatrix}0.5 & 0.1 & 0.1 & 0.3\end{pmatrix}$$
$$V_2 = \begin{pmatrix}0.7 & 0.2 & 0.1\end{pmatrix}$$

Now, since sample 4 is missing from view 2, so we have considered only $1/2$ as its probability value. Therefore,

$$V_{uni} = \begin{pmatrix}\frac{0.5\times0.7}{0.5\times0.7+(0.1+0.1+0.3)\times(0.2+0.1)} & \frac{0.1\times0.2}{0.1\times0.2+(0.5+0.1+0.3)\times(0.0.7+0.1)} & \frac{0.1\times0.1}{0.1\times0.1+(0.5+0.1+0.3)\times(0.7+0.2)} & \frac{0.3}{2}\end{pmatrix}$$
$$= \begin{pmatrix}0.7 & 0.027 & 0.012 & 0.15\end{pmatrix}$$

Again after the adjustments, the new probability is:

$$V_{uni} = \begin{pmatrix}0.7275 & 0.0545 & 0.03950.15\end{pmatrix}$$

Intuitively, we can see when both the views provide high probability, then the probability after conflation is high. Similarly, we can see that although sample 4 occurs in V_1 but it has second highest probability compared to sample 2 & 3. This is intuitive because both samples 2 & 3 have low probabilities in both the views, so the final probability score is less. Further, we have divided the probability of sample 4 with 2 because there are 2 views and it is present in only one view.

3 view settings

Let us assume there are 3 views, V_1, V_2 and V_3, having the following probability distribution:

$$V_1 = \begin{pmatrix}0.2 & 0.1 & 0.4 & 0.3\end{pmatrix}$$
$$V_2 = \begin{pmatrix}0.1 & 0.8 & & 0.1\end{pmatrix}$$
$$V_3 = \begin{pmatrix}0.2 & 0.8\end{pmatrix}$$

Now sample 3 is present only in V_1 and sample 4 is absent in V_3.

The unified probability is calculated as follows:

$$V_{uni} = \begin{pmatrix}\frac{0.2\times0.1\times0.2}{0.2\times0.1\times0.2+(0.1+0.4+0.3)\times(0.8+0.1)\times0.8} & \frac{0.1\times0.8\times0.8}{0.1\times0.8\times0.8+(0.2+0.3+0.4)\times(0.1+0.1)\times0.2} & \frac{0.4}{3} & \frac{0.3\times0.1}{0.3\times0.1+(0.1+0.2+0.4)\times(0.8+0.1)}\end{pmatrix}$$
$$= \begin{pmatrix}0.068 & 0.64 & 0.133 & 0.045\end{pmatrix}$$

After adjustment the final, V_{uni}, is :

$$V_{uni} = \begin{pmatrix}0.0965 & 0.6685 & 0.1615 & 0.0735\end{pmatrix}$$

From the unified probability we can see that sample 2 has received the highest probability value as two of the views have shown highest confidence values on sample 2. Although sample 1 is present in all the views but it has received the lowest value as it has low probability scores from all the views. As sample 3 occurs in only one view, so we have divided it's value by 3 (total 3 views are there). For sample 4, we have used the values from the views where it has occurred. Similar process is followed for combining higher order views.

There is another case, that if a view has an example which is not present in any other view then it will have an extra row which is not present in another other probability matrix. For that row, we kept the row as it is in the unified probability matrix. Because this row is giving the information about the probability of reaching other points from that missing point.

After obtaining the P_{uni} matrix we symmetrized it using the Equ.10 (in the main file).

Table 1. Description of the data sets used.

data sets	USPS	Cora	BBC	VOC
#samples	2000	2708	2012	5619
#views	2	2	2	2
#clusters	10	7	5	20
#features	76+216	2708+1433	6838 +6790	3990+0512

Experiments

Data Sets

Under this section, we have described the data sets that are used for the purpose of experimentation.

1. *US Postal Service:* The dataset contains handwritten numerals ('0'–'9'); features extracted from a collection of Dutch utility maps (https://archive.ics.uci.edu/ml/datasets/Multiple+Features). There are 2000 samples, divided into 10 groups with each group having 200 samples. In accordance with[1], for the experiment, we have used 76 Fourier coefficients of the character shapes as the first view and 216 profile correlations as the second views.

2. *Cora Data Set:* It is a document data set (http://lig-membres.imag.fr/grimal/data.html) having 2708 documents categorized into seven classes. For the purpose of experiment, we have used content and citations as two views. The features of the content view are represented by binary word vector (0/1), indicating the absence/presence of the corresponding words.

3. *BBC:* This data set consists of BBC and BBCSport corpora (http://mlg.ucd.ie/datasets/segment.html). It is a synthetic multi-view data set constructed in line with[2]. It has 2012 examples, categorized into 5 classes. The two views have dimensions of 6838 and 6790, respectively.

4. *VOC 2007[3]:* It consists of total 9963 images, divided into 20 categories (http://host.robots.ox.ac.uk/pascal/VOC/voc2007/index.html). In our experiment, we have used two views, i.e., 512 dimensional GIST features and 399 dimensional tag features. Multi-labeled and unlabeled images are removed from the database, only 5619 examples are used for final evaluation as done in[2].

Comparison Methods

Under this section, we have discussed about the algorithms that were used for comparison. The details are as follows:

1. *CCA:* The canonical correlation analysis is used to generate the low-dimensional unified embedding and then K-means is applied on the embedding to generate the clusters.

2. *CentroidSC*[1]: Here the spectral embeddings of all the views are regularized to be similar to a unified spectral embedding and then K-means is applied on the unified embedding.

3. *PairwiseSC*[1]: The spectral embeddings of all the views are regularized to be similar and then K-means is applied on one of the embeddings.

4. *Partial multi-View Clustering (PVC)*[4]: The NMF-based method in used to deal with complete and incomplete multi-view clustering.

5. *Robust Multi-View Spectral Clustering (RMSC)*[5]: A unified transition matrix is learned based on sparse and low-rank constraints, and finally spectral analysis is used for final clustering.

6. *Multiview Triplets Embedding (MultiTE)*[2]: It generates a low-dimensional embedding by exploiting the similarity triplets calculated from different views.

7. *MvNE+MOO:* Our proposed multi-view clustering methodology uses conflation method to combine the views in the probabilistic domain and generates a unified embedding. We applied multi-objective optimization algorithm, AMOSA[6], on the embedded data sets to obtain the clusters. AMOSA automatically determines the clusters from the data set. From the Pareto-front we have reported the results of the solution which have high NMI values.

8. *MvNE+kmeans:* As a baseline, this method is developed. It is similar to *MvNE+MOO* but the difference is that after the generation of embedding, instead of AMOSA, single objective clustering algorithm like K-means is applied. It is done to make a fair comparison with the embedding methods which apply K-means on the embedded data set.

9. *SingleNE:* As another baseline method, we have applied *MvNE+kmeans* on every single view and reported the results of the best performing view amongst all the views.

10. *AvgMvNE:* As a baseline method, we have at first generated a probability distribution of the samples on each view and then combined the distributions by calculating the average of the probabilities over the views. Final embedding is generated by minimizing the KL divergence between the obtained average probability and the probability in embedded domain.

Experimental Settings

For CentroidSC, PairwiseSC, PVC, and RMSC methods, we have used the codes released by corresponding authors. For our method, empirically we have selected the size of low dimensional embedding, dim, as 30 for all data sets. The size of the nearest neighbour, k, is set to 50 empirically for all data sets. As for K-means, we have used the python package scikit-learn[7], with *n_init* parameter value sets to 20 and other parameters set to default values except for the $n\_clusters$ parameter, which is the number of clusters, changed accordingly with the data set. The total number of iterations of gradient descent is set to 2000, initial momentum is set to 0.5 and the final momentum is set to 0.9. Initially learning rate(η) is set to 200 and after every iteration it is updated by adaptive learning rate scheme described by Jacobs et al.[8].

Results and Discussion

Results for complete multi-view clustering

In Table 2 and 3, we have compared the NMI and accuracy (ACC) values over six datasets obtained by different clustering methods. In terms of NMI and ACC metrics, our proposed methodology, *MvNE+MOO*, shows an improvement of $1.5\% - 2.1\%$ and $1.2\% - 2.5\%$ for all the data sets over state-of-the-art algorithms. Due to added advantage of MOO based clustering, our proposed algorithm, *MvNE+MOO*, outperforms all other algorithms.

Intuitively, we can see MultiTE and MvNE work on the similar principle of preserving the neighbourhood identity to obtain the embedding. They minimize the distances between the similar points (neighbouring points) and maximize the distances between the non neighbouring points. Hence, our baseline algorithm, *MvNE+kmeans*, produces similar results compared to MultiTE. However, for two data sets, CCV and Cora, MultiTE outperforms our *MvNE+kmeans* by 0.06% and 0.02%. respectively, in terms of NMI. The differences are not significantly high.

PairwiseSC and CentroidSC methods perform spectral analysis on the Laplacian matrices from multi-view data to generate the embeddings, which use pairwise similarities of the examples.

RMSC also performs clustering in a similar manner with CentroidSC and PairwiseSC. On the other hand, our proposed method *MvNE* obtains the unified embedding by preserving the probability distribution of the points in the embedded dimension and also retains their neighbourhood identity in the embedded domain. It is easy to obtain and also reveals the true structure of the clusters accurately compared to other methods.

PVC generates unified low-dimensional embedding by using NMF. Its application is limited to multi-view data with negative features because of the usage of NMF.

The baseline method, *SingleNE*, generates low-dimensional embedding for each view individually and returns the embedding of the best view.

The baseline method, *AvgMvNE*, performs poorly compared to most of the state-of-the-art and all baseline methods. Here, the probability distributions of the points across the views are combined by averaging them.

Results for Incomplete Multi-view Clustering

Incomplete view data sets are constructed with the settings similar to[4]. The settings are as follows:

1. First setting: In this case, all the views are incomplete, i.e., they have loss of information. That means some samples occur only in one of the views, either view 1 or view 2.

2. Second setting: Under this setting, at least one view is complete, i.e., it has complete information. It means all the samples are present in at least one of the views and the other views have some missing samples.

For the two settings, we randomly select $10\% - 90\%$ examples, with 20% examples appearing only in one view. The entire process is repeated for 20 times and the average values are reported.

Table 2. RESULTS ON THE SIX DATABASES IN TERMS OF NMI. STANDARD DEVIATION IS ALSO REPORTED.

NMI(%)	BBC	USPS	CCV	VOC	3sources	Cora
CCA	16.08(7.29)	74.87(3.02)	23.57(1.02)	46.24(4.66)	59.87(6.02)	1.12(0.43)
CentroidSC	73.12(3.65)	74.12(2.28)	21.84(0.63)	53.08(1.04)	62.36(2.41)	24.41(1.24)
PairwiseSC	73.54(3.98)	73.36(1.32)	19.43(0.37)	51.27(1.30)	61.95(2.66)	28.08(2.04)
PVC	NA	68.04(4.84)	18.51(0.72)	65.72(3.02)	68.45(0.03)	23.36(1.53)
RMSC	74.65(3.54)	71.73(1.16)	21.51(0.73)	56.01(0.95)	62.14(2.29)	14.57(1.37)
MultiTE	81.58 (0.60)	82.32 (0.53)	25.24 (0.26)	67.85 (0.44)	79.36 (1.95)	39.75 (0.25)
SingleNE	64.02(1.01)	67.16(0.45)	19.78(0.56)	63.53(0.49)	70.22(0.97)	33.28(0.38)
AvgMvNE	60.12(0.61)	62.16(0.15)	18.51(0.32)	61.43(0.24)	55.12(0.71)	21.29(0.14)
MvNE+kmeans	82.06 (0.51)	82.52 (0.59)	25.18 (0.18)	68.02 (0.41)	79.56 (1.80)	39.73 (0.21)
MvNE+MOO	83.11 (0.15)	83.61 (0.41)	27.08 (0.27)	69.72(0.57)	81.46 (0.74)	40.67 (0.29)

Table 3. RESULTS ON THE SIX DATABASES IN TERMS OF ACCURACY(ACC). STANDARD DEVIATION IS ALSO REPORTED.

ACC(%)	BBC	USPS	CCV	VOC	3sources	Cora
CCA	35.41(8.46)	74.51(4.87)	27.02(2.12)	41.45(6.23)	62.24(6.45)	26.85(1.60)
CentroidSC	87.19(2.01)	77.74(5.87)	25.38(0.90)	56.42(2.03)	58.37(3.28)	43.21(0.98)
PairwiseSC	85.75(8.97)	75.86(5.74)	23.45(0.48)	51.66(1.74)	58.35(3.24)	44.56(3.54)
PVC	NA	67.64(4.70)	18.24(0.74)	65.72(3.12)	68.37(0.06)	42.03(1.35)
RMSC	89.08(6.02)	78.87(4.95)	25.74(0.98)	51.64(3.08)	58.20(3.51)	33.72(2.38)
MultiTE	93.74(0.99)	85.96(1.45)	26.88(0.61)	69.97(1.03)	82.92(2.64)	59.96(0.86)
SingleNE	84.87(0.12)	64.71(0.47)	22.29(0.61)	57.64(0.11)	79.11(1.02)	46.84(0.73)
AvgMvNE	64.71(0.07)	62.51(1.24)	21.57(0.35)	52.10(1.41)	74.12(1.32)	44.35(0.54)
MvNE+kmeans	93.89(1.02)	86.01(1.35)	26.78(0.59)	70.03(1.04)	82.84(1.58)	60.02(0.84)
MvNE+MOO	94.54(0.24)	88.21(0.98)	27.89(0.21)	72.01(1.01)	84.14(0.52)	61.04(0.84)

Table 4. THE P-VALUES OBTAINED BY ONE-WAY ANOVA TEST on COMPARING *MvNE+MOO* WITH OTHER METHODS IN TERMS OF NMI.

	BBC	USPS	CCV	VOC	3sources	Cora
CCA	0.000410	0.000174	0.000071	0.001451	0.000462	0.0002856
CentroidSC	0.001109	0.000047	0.002305	0.000946	0.000858	0.000153
PairwiseSC	0.0008751	0.006615	0.000024	0.000661	0.000058	0.000445
PVC	NA	0.000046	0.000108	0.000765	0.000068	0.000642
RMSC	0.000090	0.002178	0.000412	0.000511	0.000835	0.003327
MultiTE	0.005418	0.001345	0.000185	0.003715	0.000067	0.000748
SingleNE	0.000844	0.000764	0.000290	0.000657	0.001179	0.001846
AvgMvNE	0.0000361	0.002712	0.000684	0.000097	0.000528	0.004970
MvNE+kmeans	0.0004185	0.00003152	0.000849	0.001054	0.0000046	0.002201

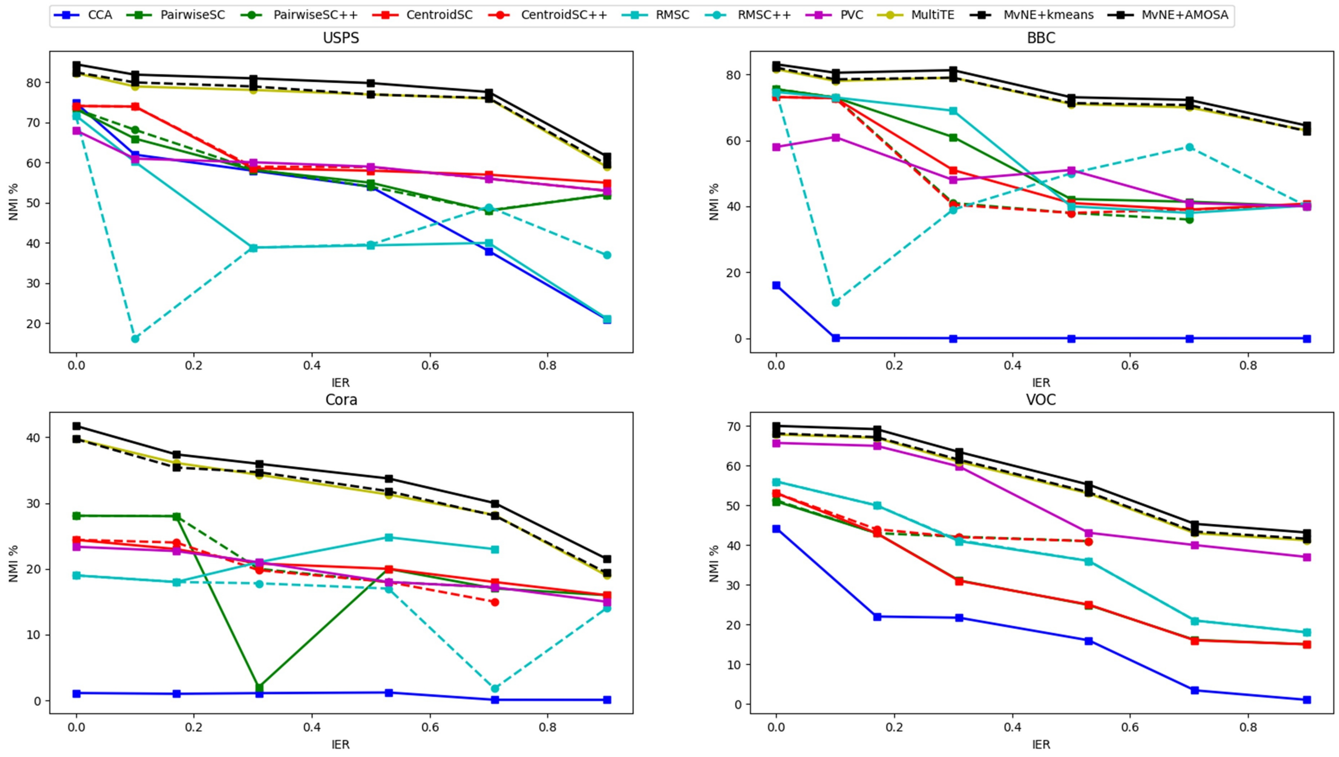

Figure 1. Change in NMI for four data sets under first setting.

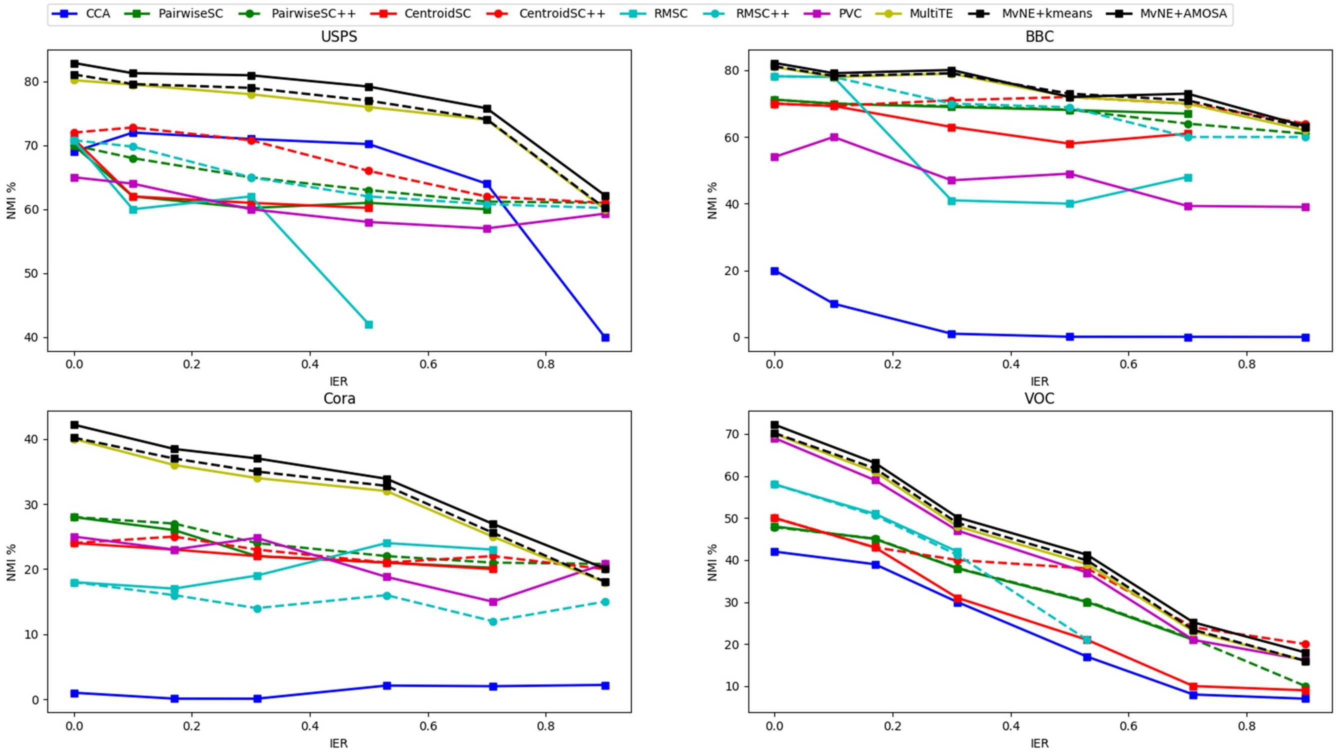

Figure 2. Change in NMI results when only first view is incomplete.

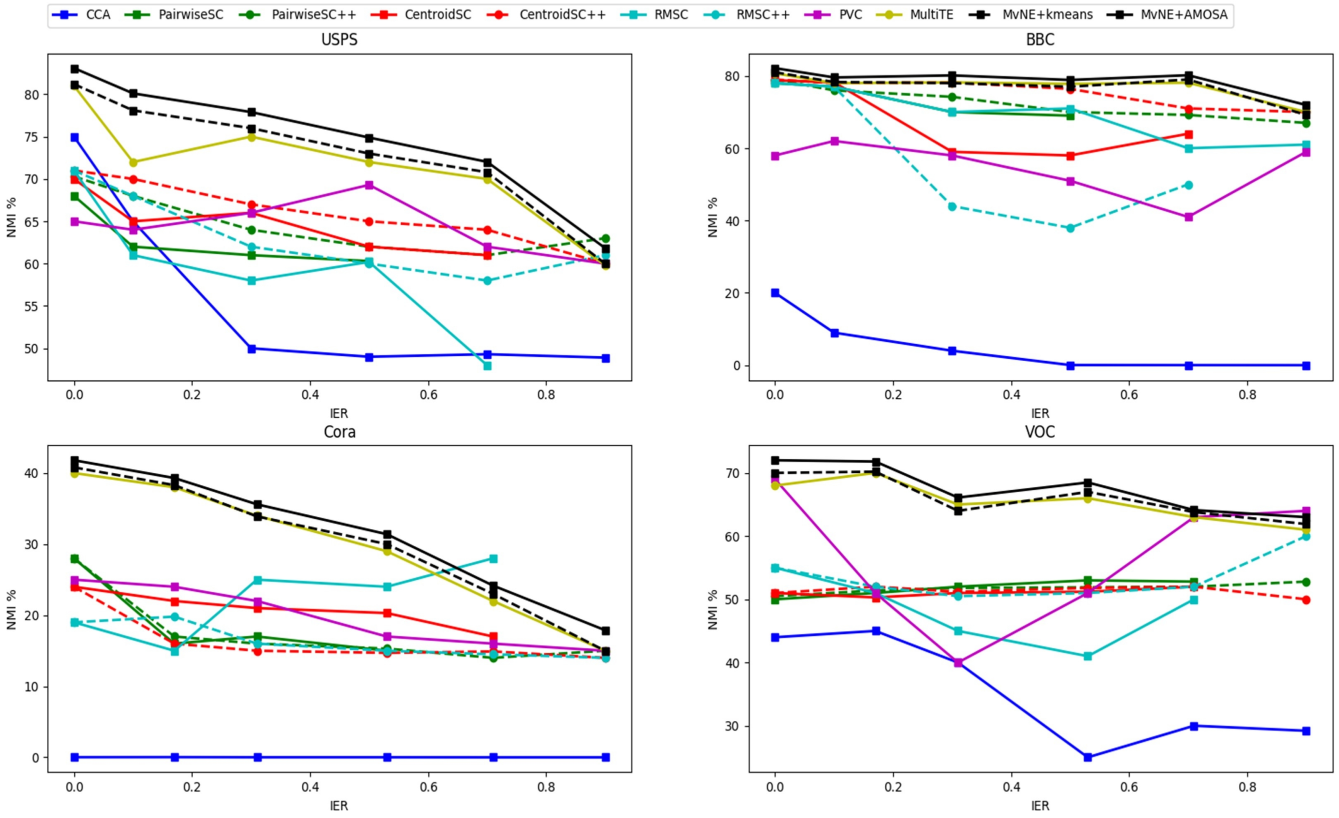

Figure 3. Change in NMI results when second view is incomplete.

For the second setting, we followed the steps proposed in[9] to preprocess the methods, CentroidSC, PairwiseSC, and RMSC. Similarly, to deal with the first setting, we followed the steps proposed in[10], to preprocess CentroidSC, PairwiseSC, and RMSC methods. The preprocessed methods are denoted as CentroidSC++, PairwiseSC++ and RMSC++.

For both the settings, we have shown results on the four data sets, USPS, Cora, BBC and VOC. It is possible to obtain similar results for other data sets.

Results Under the First Setting

In Fig.1, we have shown the NMI values obtained by all clustering methods under first setting for four data sets, i.e., BBC, USPS, Cora and VOC. The relative number of examples appearing only in a single view is denoted by incomplete example ration (IER). Overall, our proposed methodology, *MvNE+MOO* outperforms other state-of-the-art and baseline methods, with different IERs.

For the spectral based methods, CentroidSC, PairwiseSC, and RMSC, we applied the proposed method in[10] to fill the kernel matrices of the incomplete views. The modified methods are represented as CentroidSC++, PairwiseSC++, and RMSC++. From the Figure 1, it is seen that the modified methods show little improvement.

In some cases, specially in RMSC, the final clustering is harmed, it may be because under incomplete views, the proposed method in[11] fails to promote kernel matrix completion. Another reason is that the parameter selection for the preprocessing methods[10] has to be performed more carefully.

As for the PVC, it uses NMF to generate embedding for incomplete multi-view data sets without pre-processing. However, as both the views are incomplete under first setting, it fails to explore the data relations effectively. Our proposed methodology, *MvNE+MOO*, outperforms this method.

For the methods not designed for incomplete-multi-view clustering, i.e., RMSC, CentroidSC, CCA and PairwiseSC, we replaced the missing features with zeros. For these methods we do not pre-process the data. From the figure it can be seen that our method degrades less compared to other methods, with increase in IER. This shows the efficiency of our algorithm to deal with incomplete multi-view data.

Results Under the Second Setting

In Fig. 2 and 3, we have shown the NMI results obtained by all the clustering algorithms on BBC, USPS, VOC and Cora data sets, when either first view or second view is incomplete. Unlike first settings, here at least one view is complete. Here also, *MvNE+Kmeans* performs better than other state-of-the-art methods and generates similar results compared to MultiTE. Our proposed method *MvNE+MOO* outperforms all the other algorithms.

From the figures, it is also seen that the modified algorithms, i.e., PairwiseSC++, RMSC++, and CentroidSC++, perform better than the original ones. It is because under second setting we have at least one complete view to guide the learning of kernel matrices of incomplete views.

Other methods perform a little better with the same IER compared to those under first setting. The reason is, there is at least one complete view under second settings which is better compared to all incomplete views under first setting.

Statistical Significance Test

For statistical significance test we have used a non-parametric test, one-way Analysis of Variance (ANOVA) because it is independent of the distribution type of the data set. The test is performed at 1% significance level. The results obtained by our proposed methodology, *MvNE+MOO*, by 20 runs are compared with other algorithms. The p-values are reported in the Table 4. The p-values reported show that the results obtained are statistically significant.

References

1. Kumar, A., Rai, P. & Daume, H. Co-regularized multi-view spectral clustering. In Shawe-Taylor, J., Zemel, R. S., Bartlett, P. L., Pereira, F. & Weinberger, K. Q. (eds.) *Advances in Neural Information Processing Systems 24*, 1413–1421 (Curran Associates, Inc., 2011).
2. Yin, Q., Wu, S. & Wang, L. Multiview clustering via unified and view-specific embeddings learning. *IEEE Transactions on Neural Networks Learn. Syst.* **29**, 5541–5553, DOI: 10.1109/TNNLS.2017.2786743 (2018).
3. Everingham, M., Van Gool, L., Williams, C. K. I., Winn, J. & Zisserman, A. The PASCAL Visual Object Classes Challenge 2007 (VOC2007) Results. http://www.pascal-network.org/challenges/VOC/voc2007/workshop/index.html.
4. Li, S.-Y., Jiang, Y. & Zhou, Z.-H. Partial multi-view clustering. In *Twenty-Eighth AAAI Conference on Artificial Intelligence* (2014).
5. Xia, R., Pan, Y., Du, L. & Yin, J. Robust multi-view spectral clustering via low-rank and sparse decomposition. In *Twenty-Eighth AAAI Conference on Artificial Intelligence* (2014).
6. Acharya, S., Saha, S. & Thadisina, Y. Multiobjective simulated annealing-based clustering of tissue samples for cancer diagnosis. *IEEE J. Biomed. Heal. Informatics* **20**, 691–698, DOI: 10.1109/JBHI.2015.2404971 (2016).
7. Pedregosa, F. *et al.* Scikit-learn: Machine learning in Python. *J. Mach. Learn. Res.* **12**, 2825–2830 (2011).
8. Jacobs, R. A. Increased rates of convergence through learning rate adaptation. *Neural networks* **1**, 295–307 (1988).
9. Trivedi, A., Rai, P., Daumé III, H. & DuVall, S. L. Multiview clustering with incomplete views. In *NIPS Workshop* (2010).
10. Shao, W., Shi, X. & Philip, S. Y. Clustering on multiple incomplete datasets via collective kernel learning. In *2013 IEEE 13th International Conference on Data Mining*, 1181–1186 (IEEE, 2013).
11. Bordes, A., Usunier, N., Garcia-Duran, A., Weston, J. & Yakhnenko, O. Translating embeddings for modeling multi-relational data. In *Advances in neural information processing systems*, 2787–2795 (2013).

Heatmaps of the datasets

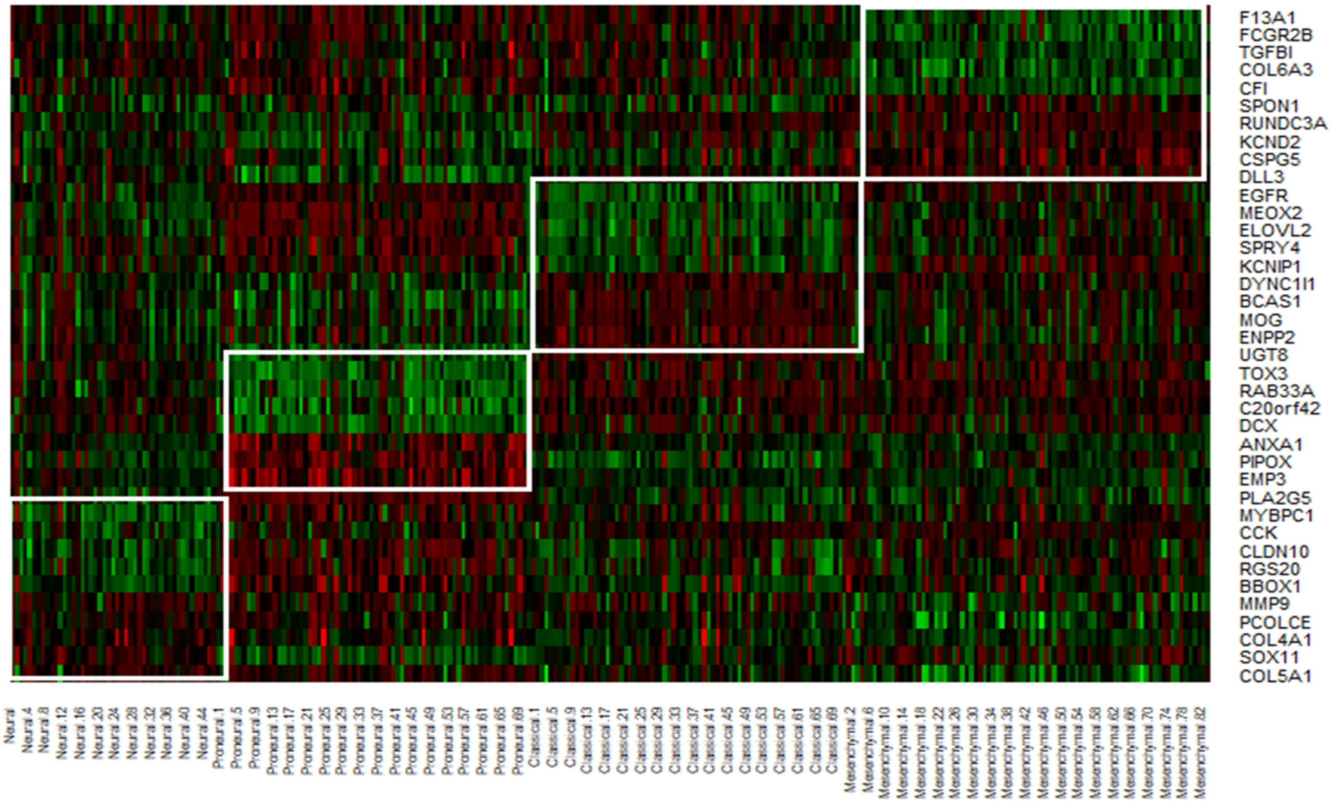

Figure 4. Heatmap of GBM

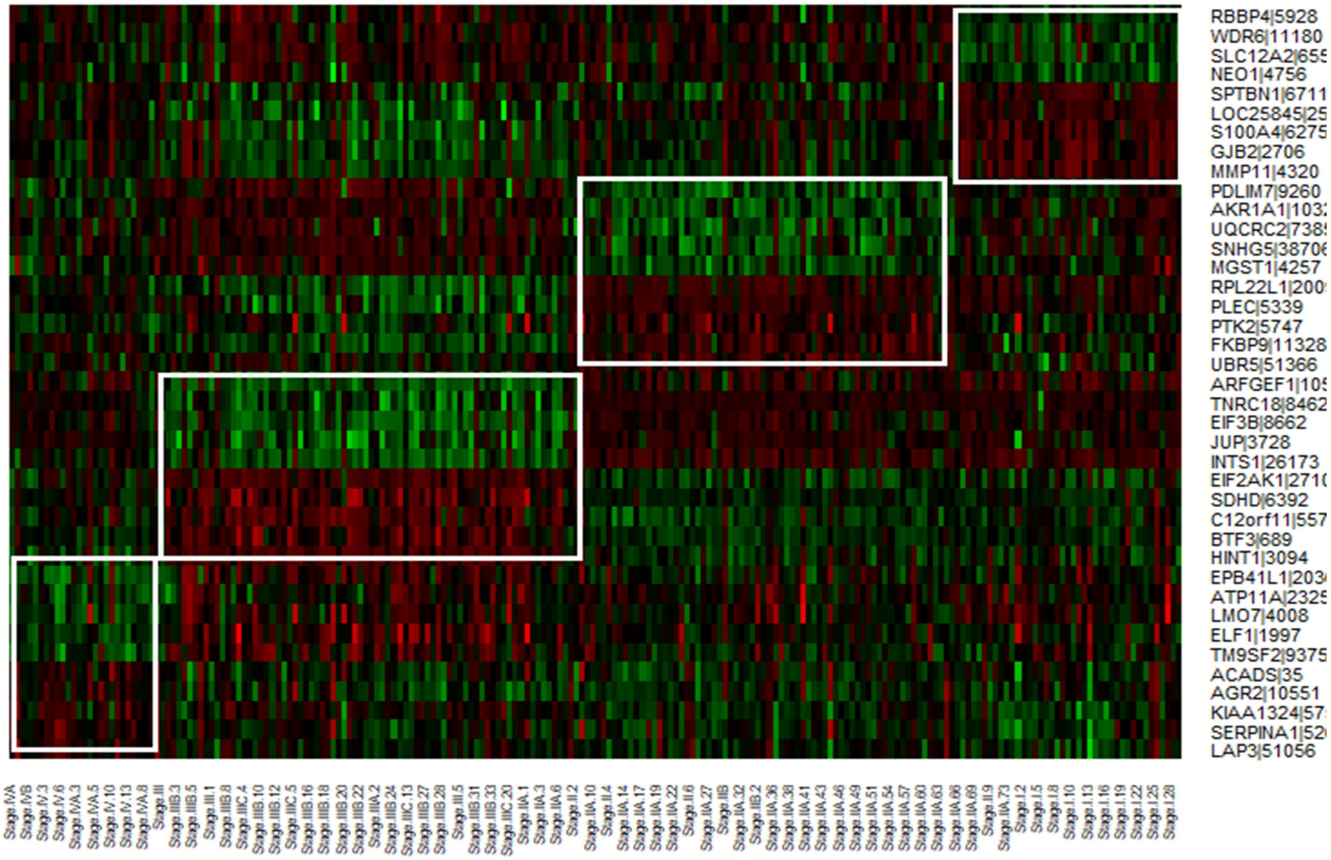

Figure 5. Heatmap of COAD

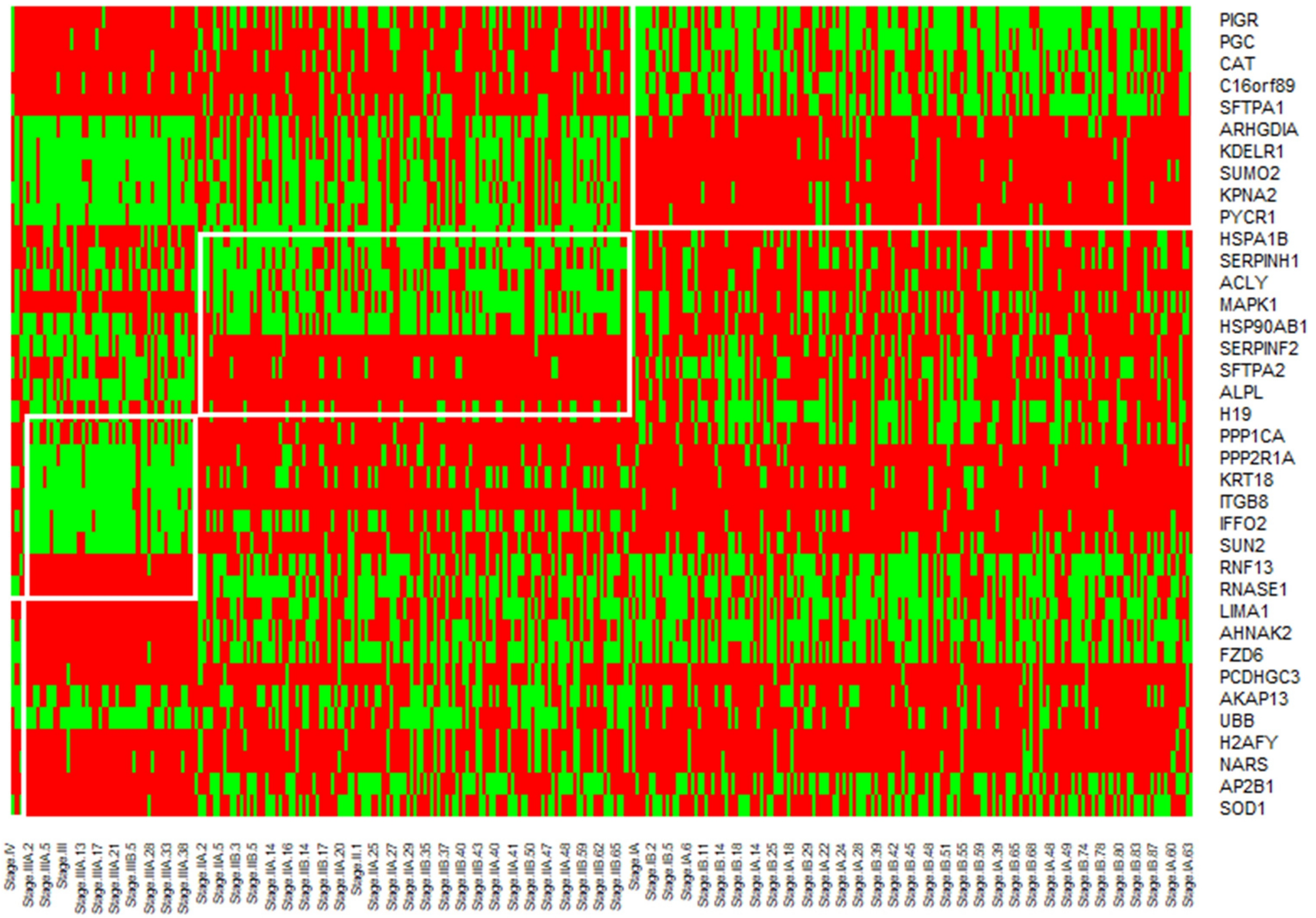

Figure 6. Heatmap of LUNG

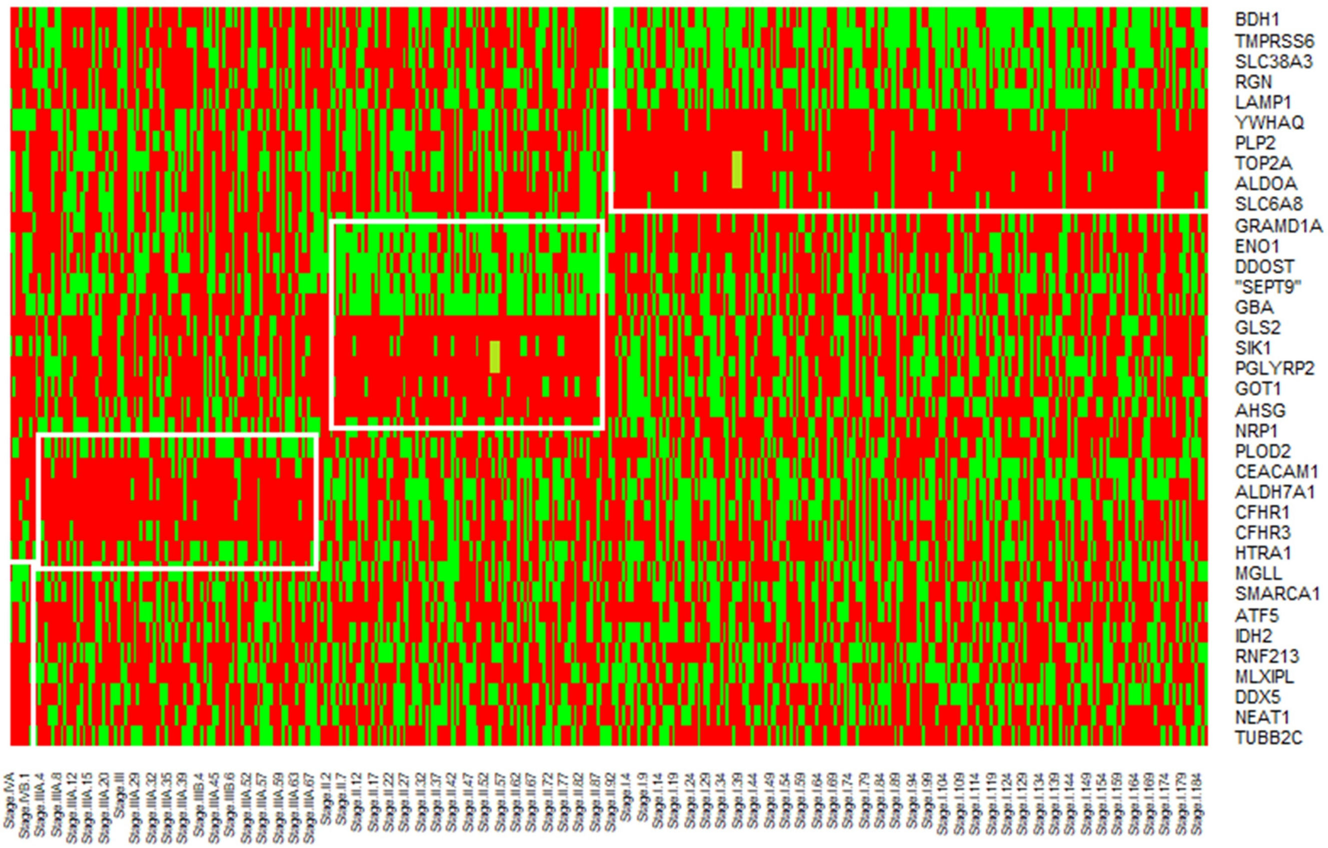

Figure 7. Heatmap of LIVER

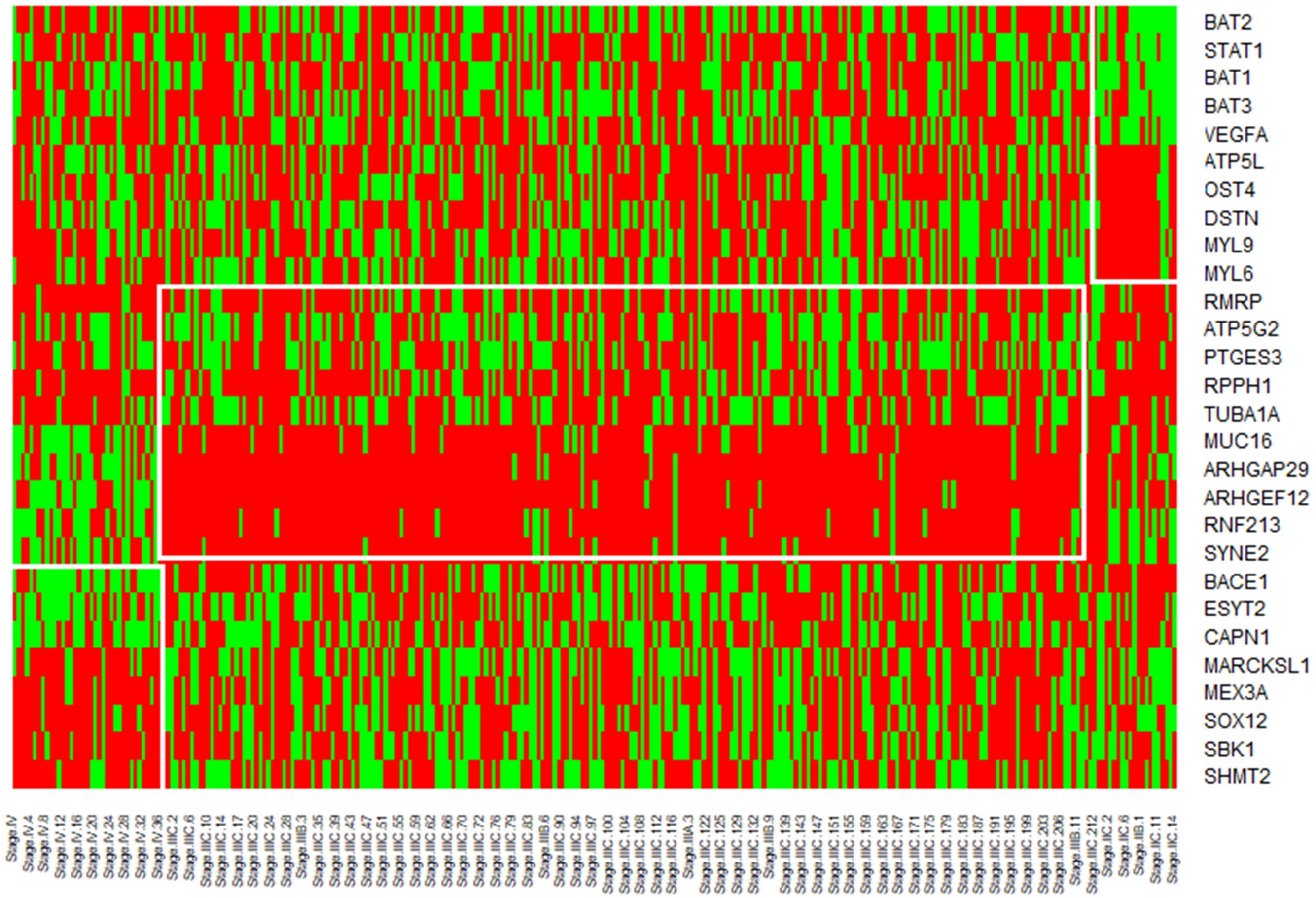

Figure 8. Heatmap of OVARIAN CANCER

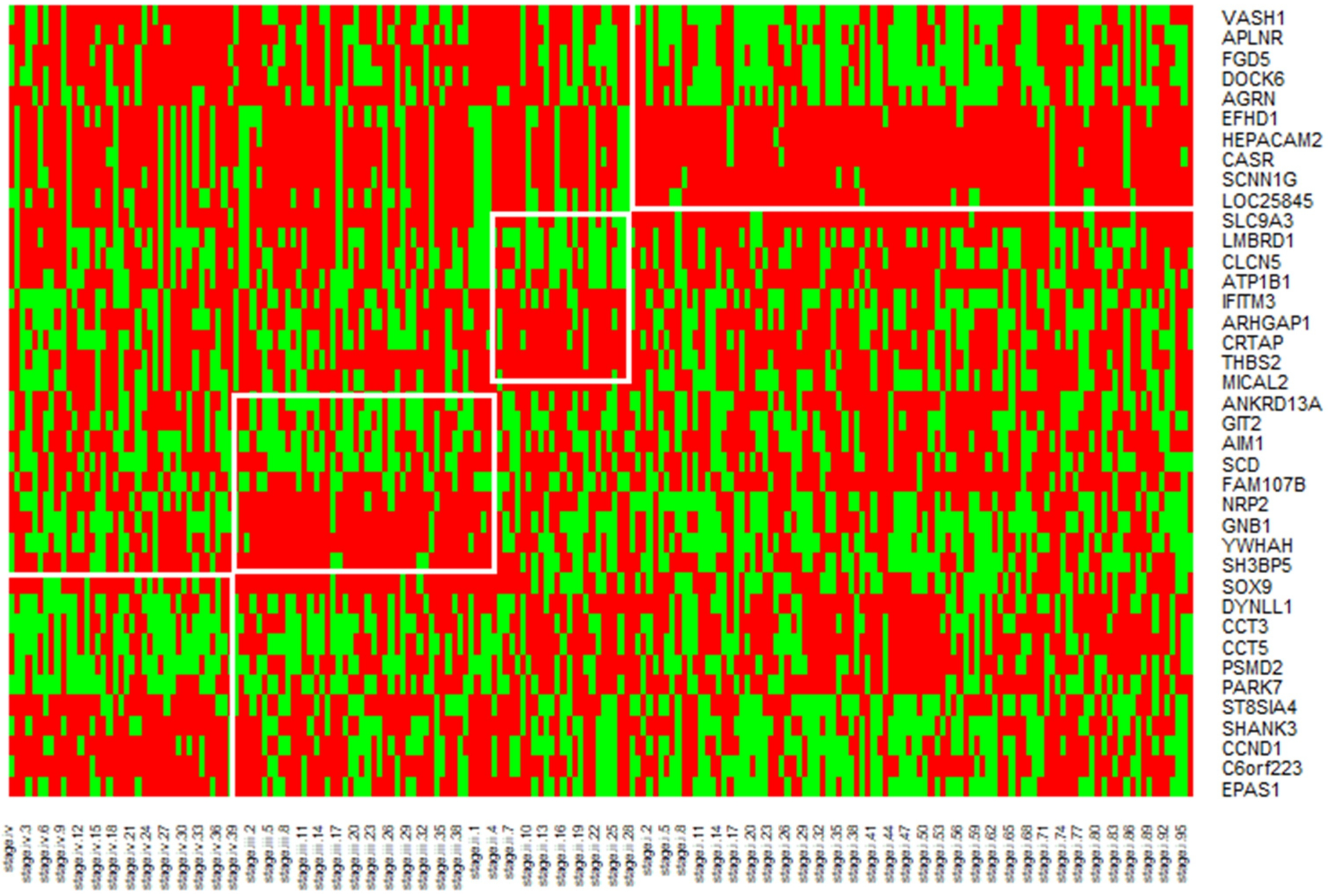

Figure 9. Heatmap of KIDNEY CANCER

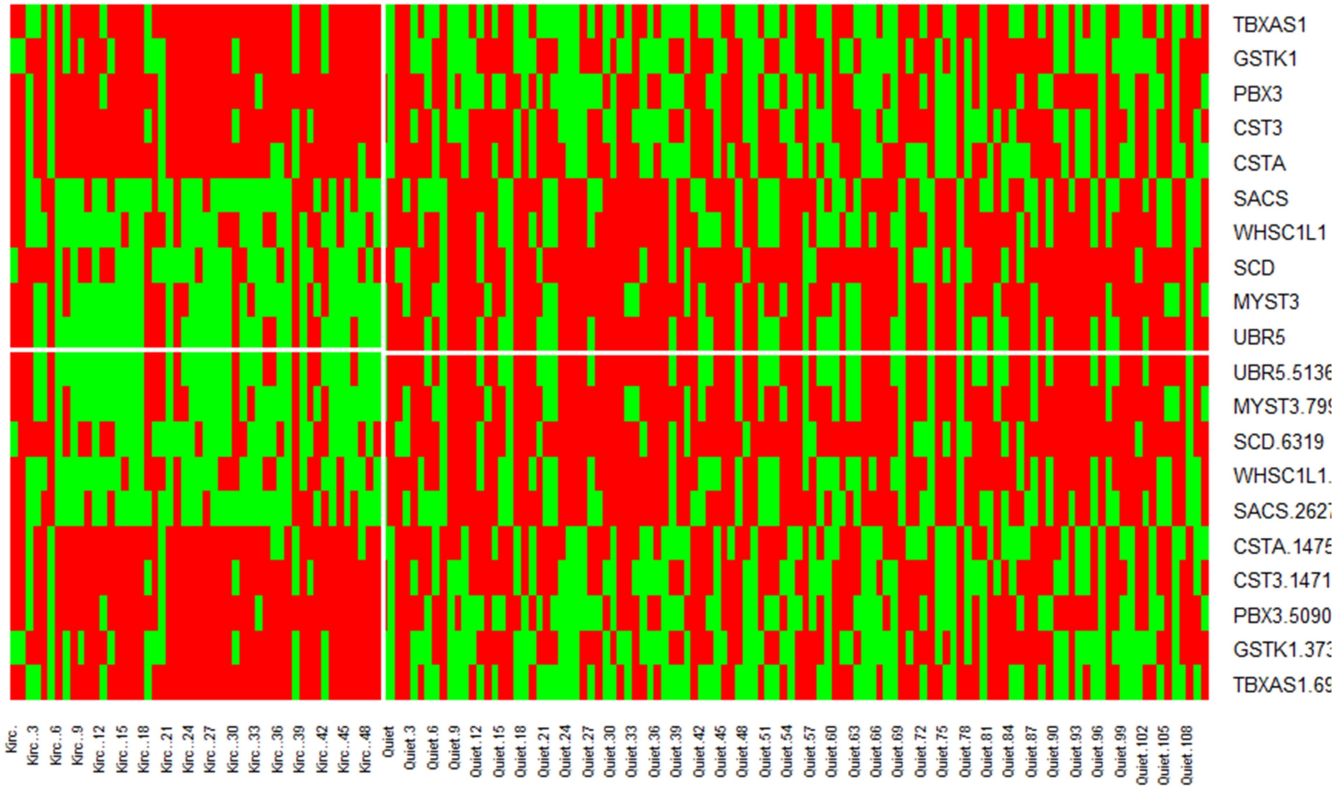

Figure 10. Heatmap of AML. Here the clusters have all the genes common. From the figure we cal see the gene up regulated in one class is down regulated in other .

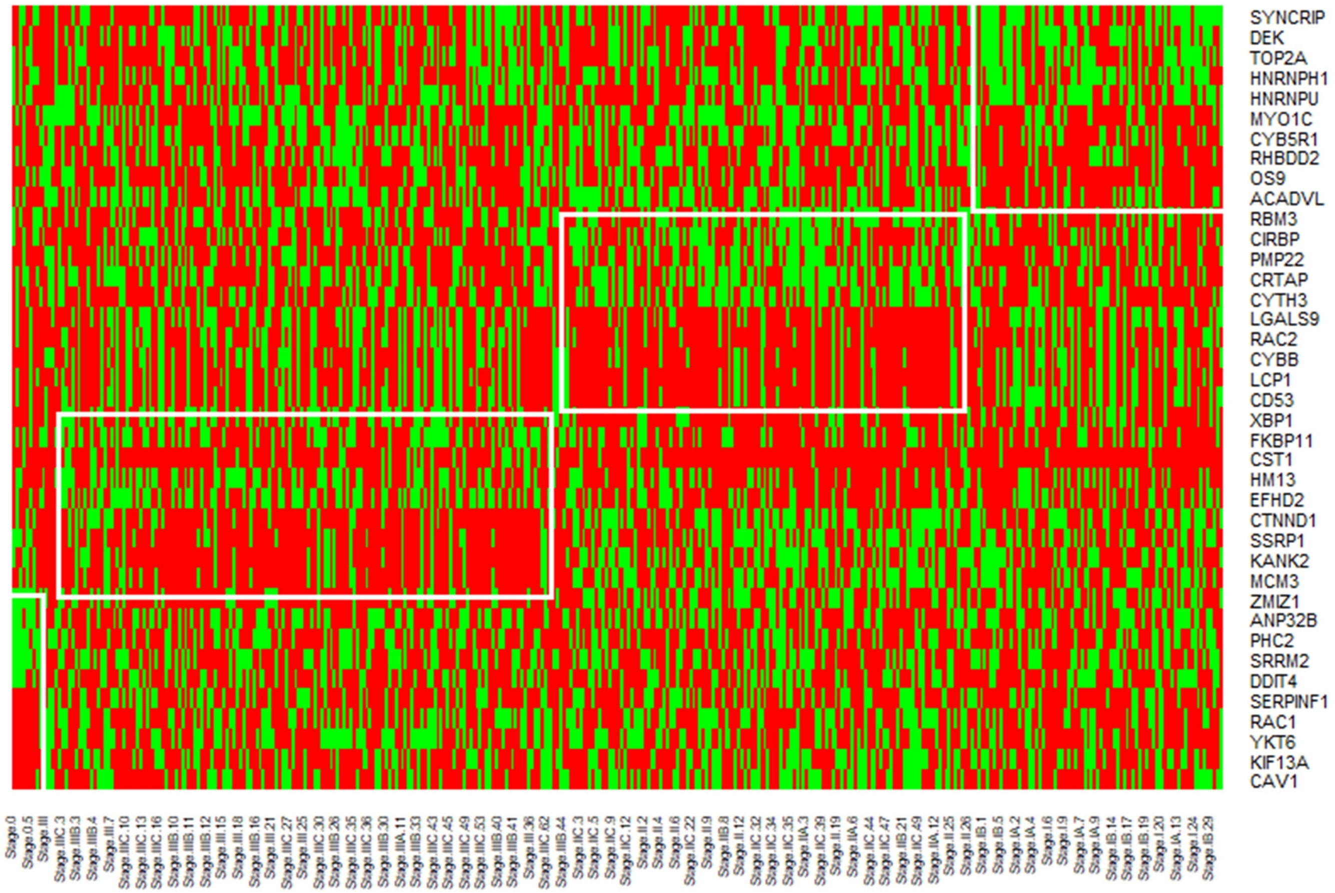

Figure 11. Heatmap of Melanoma

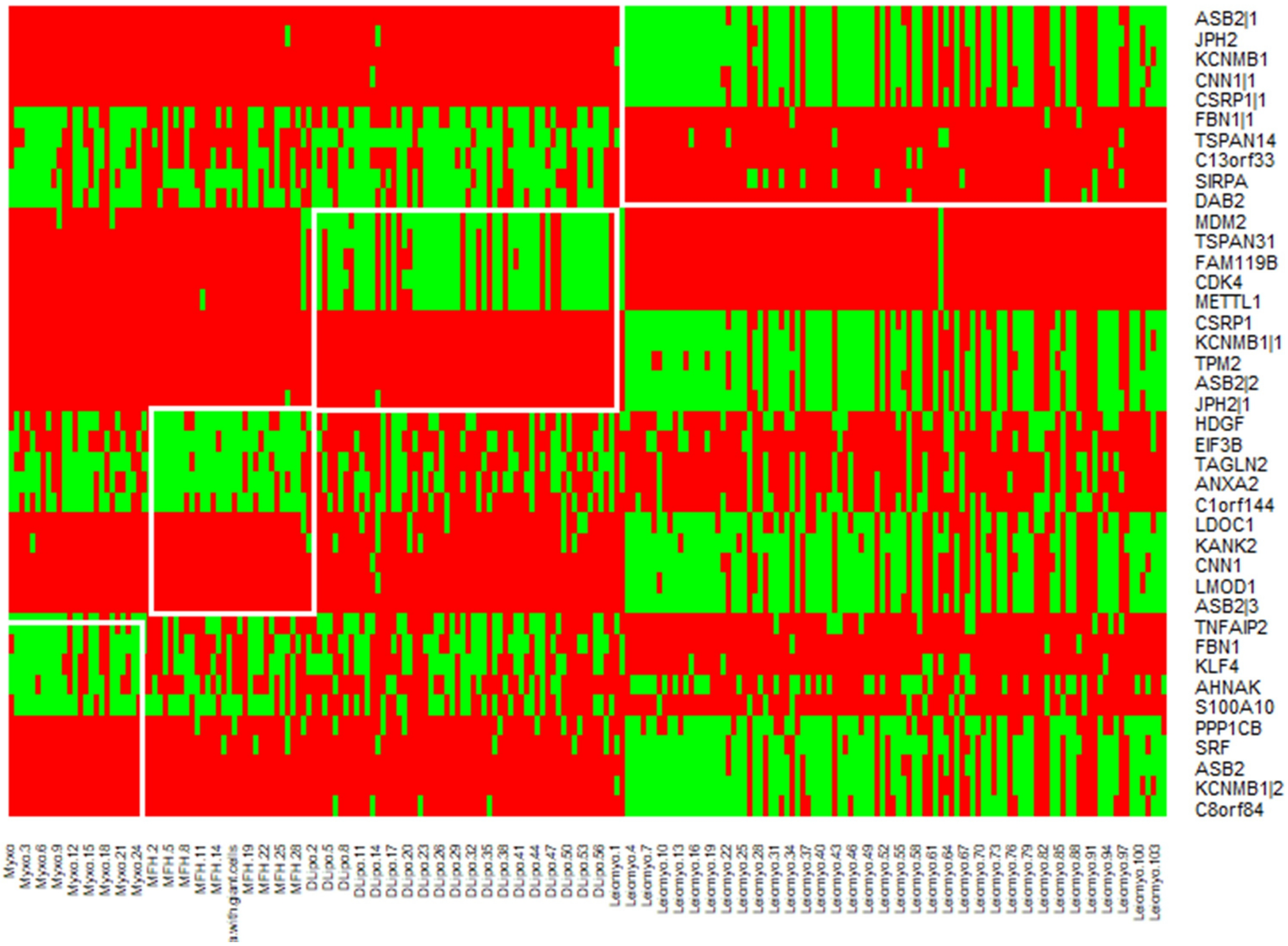

Figure 12. Heatmap of Sarcoma. There are 9 duplicate genes.Here we kept the duplicate genes.
